# Combinatorial mapping of E3 ubiquitin ligases to their target substrates

**DOI:** 10.1016/j.molcel.2025.01.016

**Published:** 2025-02-06

**Authors:** Chase C. Suiter, Diego Calderon, David S. Lee, Melodie Chiu, Shruti Jain, Florence M. Chardon, Choli Lee, Riza M. Daza, Cole Trapnell, Ning Zheng, Jay Shendure

**Affiliations:** 1 Molecular and Cellular Biology Program, University of Washington, Seattle, WA 98195, USA; 2 Department of Genome Sciences, University of Washington, Seattle, WA 98195, USA; 3 Seattle Hub for Synthetic Biology, Seattle, WA 98195, USA; 4 Brotman Baty Institute for Precision Medicine, University of Washington, Seattle, WA 98195, USA; 5 Allen Discovery Center for Cell Lineage Tracing, Seattle, WA 98195, USA; 6 Department of Pharmacology, University of Washington, Seattle, WA 98195, USA; 7 Howard Hughes Medical Institute, Seattle, WA 98195, USA; 8 Lead contact

## Abstract

E3 ubiquitin ligases (E3s) confer specificity of protein degradation through ubiquitination of substrate proteins. Yet the vast majority of the >600 human E3s have no known substrates. To identify proteolytic E3-substrate pairs at scale, we developed COMET (COmbinatorial Mapping of E3 Targets), a framework for testing the role of many E3s in degrading many candidate substrates within a single experiment. We applied COMET to SCF ubiquitin ligase subunits that mediate degradation of target substrates (6,716 F-box-ORF combinations) and E3s that degrade short-lived transcription factors (TFs) (26,028 E3-TF combinations). Our data suggest many E3-substrate relationships are complex rather than 1:1 associations. Finally, we leverage deep learning to predict the structural basis of E3-substrate interactions, and probe the strengths and limits of such models. Looking forward, we consider the practicality of transposing this framework, *i.e.* computational structural prediction of all possible E3-substrate interactions, followed by multiplex experimental validation.

## In Brief

The substrate(s) of most E3 ubiquitin ligases remain unknown. Suiter et al. present COMET, a combinatorial framework for identifying proteolytic E3-substrate relationships at scale. Deep learning-based structural prediction provides *in silico* validation of COMET-linked E3-substrate pairs, and a controlled assessment of computed interactions brings computational substrate discovery into focus.

## Graphical Abstract

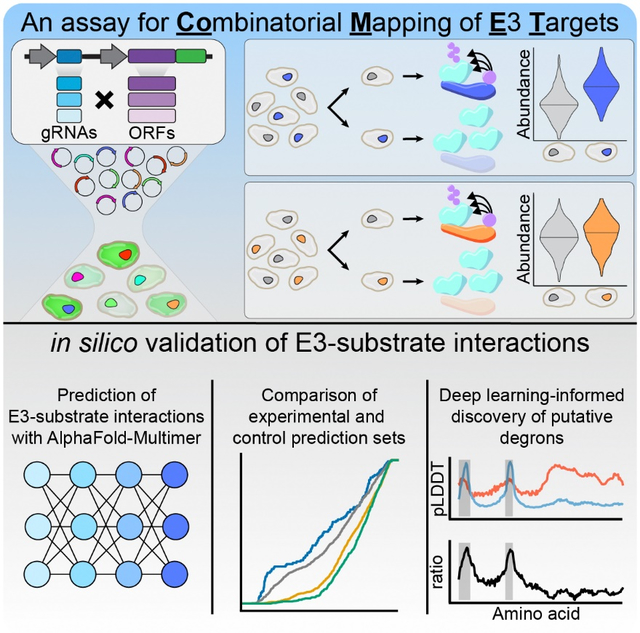

## Introduction

Rapid and precise control of cellular protein levels permit cells to regulate their homeostatic state^1^ and to respond to changing environments^2^. Such control of intracellular protein degradation is mediated by the ubiquitin-proteasome system (UPS). Ubiquitination is a post-translational covalent modification that can serve roles in transcription, DNA repair and signaling. However, it is most deeply understood as a mark for degradation by the UPS. The UPS mediates protein degradation via E3 ubiquitin ligases (E3s), which provide specificity of degradation by selecting specific substrate proteins^3^. Although there are >600 human E3s^4^, the target substrate(s) for the vast majority of E3s remain unknown, partly due to the challenging biochemistry and limited scalability of methods for assessing E3-substrate pairs.

E3-substrate interactions are dynamic and substrates may be rapidly degraded upon ubiquitination^5,6^. Some high-throughput methods to identify E3 targets have been developed, most notably Global Protein Stability (GPS) profiling^7^, which leverages a dual-fluorescent reporter to quantify protein abundance on a proteome-wide scale. GPS has been applied to identify substrates of the cullin-RING ligase (CRL) family of E3s, multi-subunit ubiquitin ligases with an interchangeable substrate receptor for modular target specificity^8^. Each GPS experiment relies on a single perturbation that broadly inhibits CRL function, *e.g.* overexpression of dominant-negative cullin fragments or drug-mediated inhibition of the entire proteolysis pathway. A consequence is that GPS lacks specificity to determine the contribution of individual CRL subunits to substrate degradation. With >600 E3s and ~20,000 possible substrates per E3, these limitations curtail the potential of GPS to comprehensively dissect the landscape of proteolytic regulation in human cells.

## Results

### A combinatorial screening method for detecting target substrates of E3 ligases

**Figure 1.**
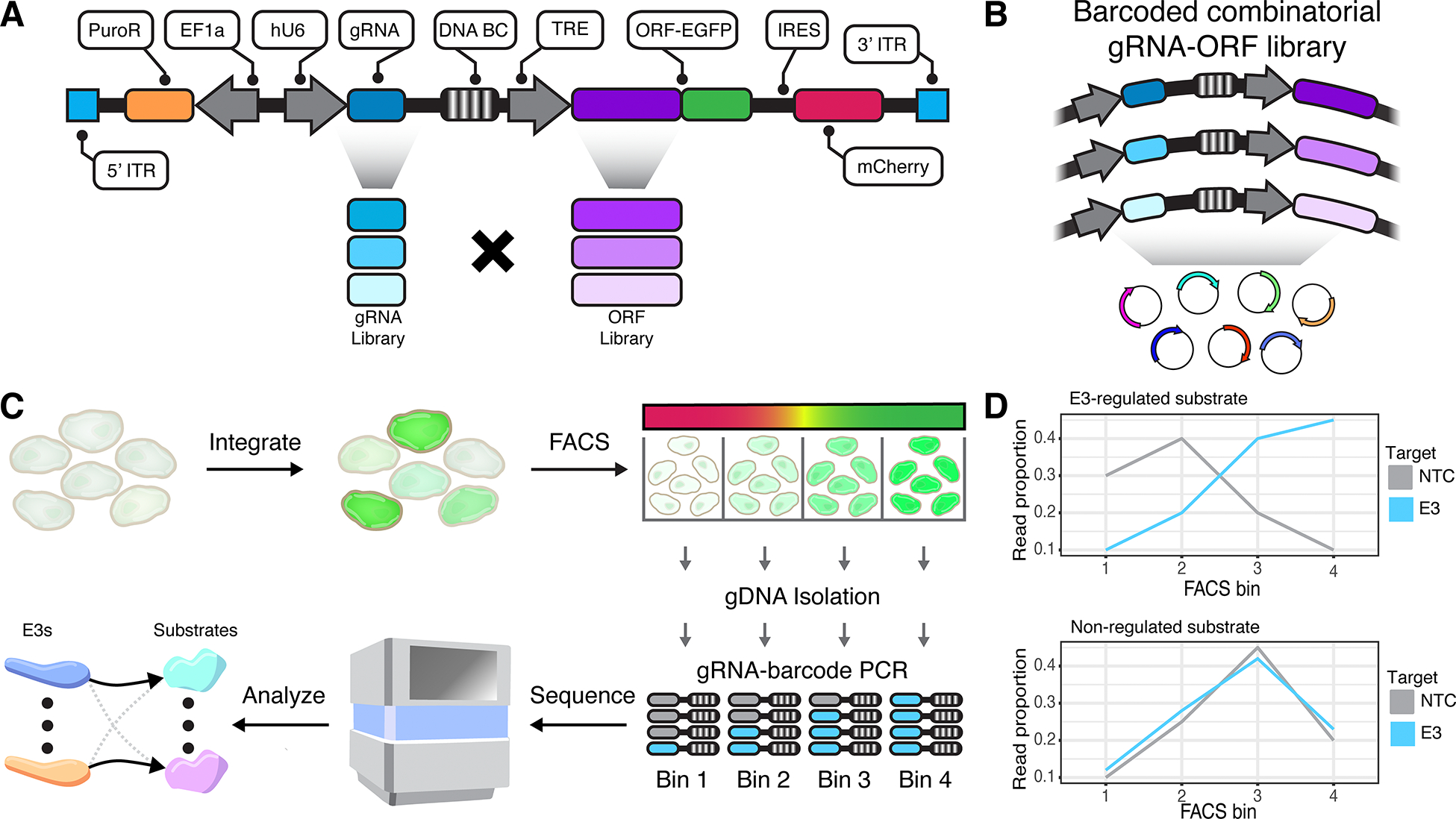
Combinatorial mapping of E3 ubiquitin ligases to their target substrates. **A)** Schematic of COMET construct. **B)** Schematic of COMET library where each plasmid represents a unique gRNA-ORF combination. **C)** Cells harboring integrated COMET libraries are sorted on the ratio of GFP:mCherry. Amplicon sequencing of gRNA-barcode pairs measures the relative abundance of perturbation-ORF pairs in each of the four bins. **D)** Illustrative distributions of read counts across 4 FACS bins for an E3-regulated substrate (top panel) and a non-regulated substrate (bottom panel).

We sought to develop a high-throughput method for screening combinatorial libraries of E3 perturbations and potential substrates. To this end, we adopted a GPS-inspired dual-fluorescent reporter^9^ expressing a GFP-fusion protein, which represents the putative ligase substrate, and an mCherry reporter translated from an internal ribosome entry site (IRES) (Figure 1A). Expression of the GFP-IRES-mCherry is controlled by the doxycycline-inducible tetracycline response element (TRE). Since the GFP-fusion and mCherry are co-expressed, the GFP:mCherry ratio, hereafter referred to as ‘protein abundance’, reflects stability of the GFP-fusion protein to degradation. To multiplex this assay, we sequentially clone DNA libraries of E3-targeting CRISPR gRNAs, human ORFs and an ORF-linked DNA barcode (Figure S1A–F). A combinatorial COMET plasmid pool contains thousands of gRNA-ORF pairs, with each pair representing a potential E3-substrate interaction (Figure 1B).

To facilitate multiplex screening, we generated monoclonal HEK293 and K562 cell lines constitutively expressing reverse tetracycline-controlled transcriptional activator (rtTA) and Cas9, hereafter termed HEK293-rtTA-Cas9 and K562-rtTA-Cas9 (Figure S1G–H). The COMET library is integrated into cells at low multiplicity of integration (MOI), such that each cell reports on the abundance of a specific ORF in the presence of a specific gRNA (Figure 1C). These cells are sorted using FACS into equal-partition bins based on the GFP:mCherry ratio. Amplicon sequencing is used to quantify gRNA-ORF pairs in each bin. For any given ORF, the read distribution across bins can be compared between cells bearing non-targeting control (NTC) vs. E3-targeting gRNAs, with differences indicating perturbation of that E3 impacts the protein-level abundance of that ORF (Figure 1D). In principle, this strategy enables many-by-many testing of E3-substrate pairs.

### Applying COMET to F-box proteins and SCF-linked substrates

**Figure 2.**
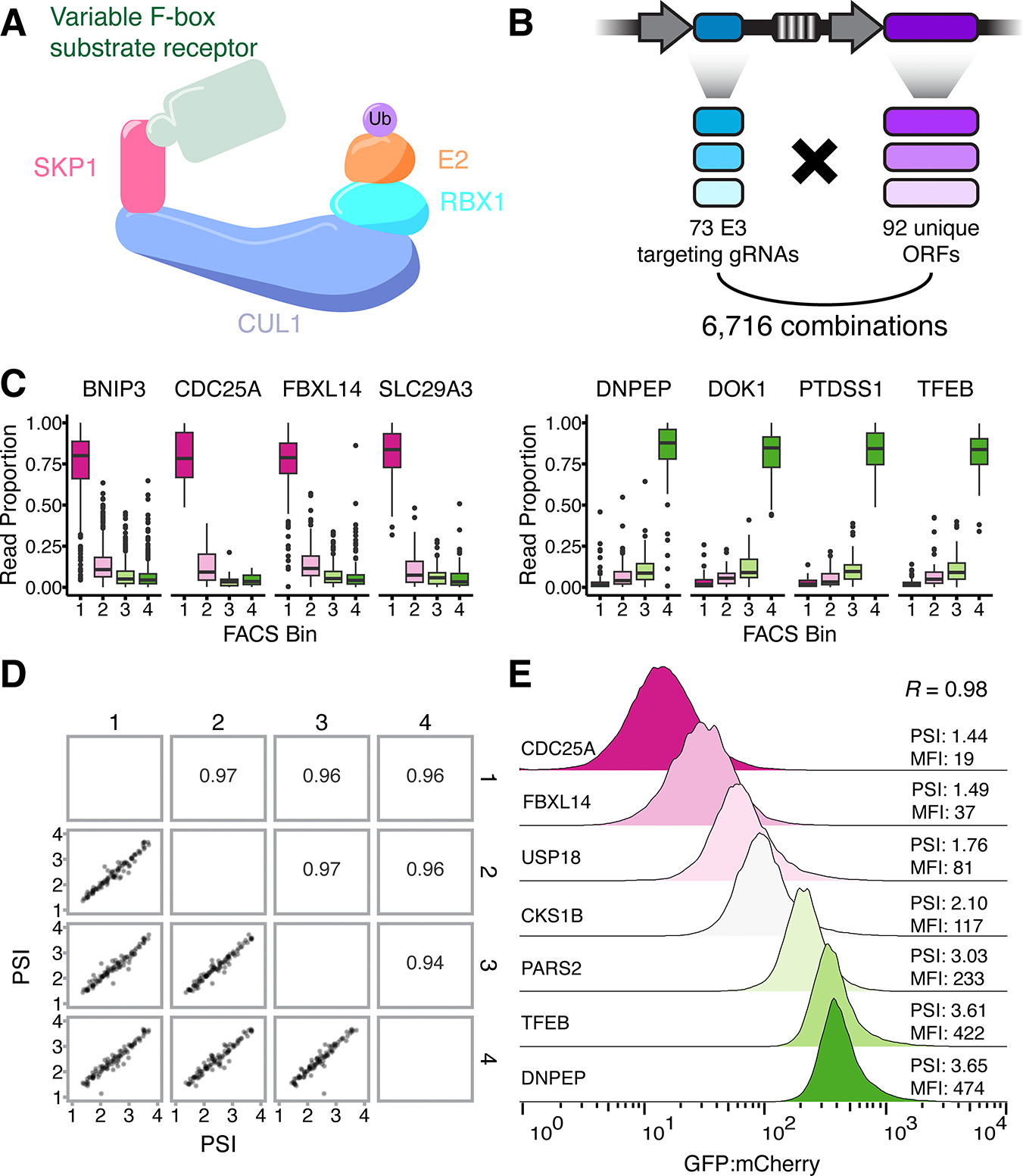
Robust measurement of baseline protein abundance with COMET. **A)** Schematic of SCF E3 ubiquitin ligase complex. **B)** Schematic of combinatorial library of F-box-targeting gRNAs and ORFs. **C)** Examples of ORFs with consistently low (left) or high (right) abundance as estimated by the distribution of their barcodes across FACS bins. **D)** Pairwise comparison of PSI between experimental transfection replicates. Pearson’s *R* shown. **E)** Validation of COMET-based PSI values with individually measured GFP:mCherry ratios for indicated proteins. Histograms display the FACS-measured GFP:mCherry ratio of cells. PSI values shown on the right are derived from COMET while MFI values reflect individually measured GFP:mCherry ratios. All data in this figure are generated from K562 cells.

For proof-of-concept, we focused on the well-characterized SCF complex^8^ (Figure 2A). The SCF is the founding member of the CRL superfamily of multisubunit E3s and is composed of a CUL1 scaffold upon which the rest of the complex is assembled. RBX1 and a ubiquitin-charged E2 bind the C-terminus of CUL1, while the N-terminus is occupied by an adaptor protein, SKP1, that mediates interactions with ~70 F-box proteins^10^. Importantly, the SCF is a modular protein degradation system. F-box proteins are interchangeable, with each recruiting a unique set of substrates for ubiquitination, thus diversifying and specifying SCF substrate ubiquitination.

To scalably identify which F-box proteins mediate degradation of which substrates, we cloned a COMET library (Figure 2B) in which each plasmid encodes: 1) an F-box-targeting gRNA, 2) a candidate substrate-GFP fusion ORF, and 3) an ORF-linked DNA barcode. We targeted 68 F-box genes^10^, core SCF components CUL1, SKP1, and RBX1, and SCF regulators NEDD8 and CAND1, with 3 gRNAs per gene, and also included 23 NTC gRNAs. For candidate substrates, we selected 30 proteins previously annotated as SCF substrates^7^, along with 62 randomly chosen proteins that were neither annotated SCF substrates nor previously tested. The 92 candidate substrates had variable endogenous expression levels in K562 cells (Figure S2A,C). The resulting library contains 6,716 F-box-ORF combinations (or 242 gRNA x 92 ORF = 22,264 barcoded constructs). Sequencing was used to generate a lookup table of barcode-ORF pairs (Table S1). 88% of the ORFs in the library had >300 barcodes, and 92% of barcodes had >90% of their reads associated with a single ORF (Figure S3A,B).

The SCF COMET library was integrated via piggyBac^11^ transposition into either HEK293-rtTA-Cas9 or K562-rtTA-Cas9 cells (day 0). Cells with integrated constructs were selected with puromycin. Expression of the ORF-GFP-IRES-mCherry reporter was induced with doxycycline on day 10, and cells were sorted into four equally partitioned bins based on the GFP:mCherry ratio on day 12. Genomic DNA was isolated from each bin, from which the gRNA-barcode region was PCR amplified and sequenced to track gRNA-ORF pair proportions across the four bins.

### Robust measurement of baseline protein abundance

To evaluate whether our assay reliably measured baseline protein abundances, we focused on data generated from K562 cells, and calculated a Protein Stability Index (PSI) as previously described^7,12^ (Table S2). The resulting PSI is a weighted average representing the mean bin position of each gRNA-ORF pair, with values ranging from 1 (maximally unstable) to 4 (maximally stable).

We first focused on ORFs paired with NTC gRNAs (*i.e.* unperturbed). The ORFs for BNIP3, CDC25A, FBXL14, and SLC29A3 paired with NTC gRNAs reliably had >75% of their reads falling in the low abundance bin (Figure 2C). In contrast, the ORFs for DNPEP, PTDSS1, TFEB, and UMPS paired with NTC gRNAs had >75% of their reads in the high abundance bin (Figure 2C). The PSIs of NTC gRNA-ORF pairs were highly reproducible (Pearson’s *R* > 0.9 for all pairwise comparisons; Figure 2D).

As COMET is a pooled experiment, we sought to validate protein abundance measurements with individually cloned ORFs. In these experiments, COMET-based PSI values correctly predicted the order of individually assayed protein stabilities (Figure 2E), and were highly correlated with mean-fluorescence intensity (MFI) of the GFP:mCherry ratio (Pearson’s *R* = 0.98; Figure S3E). Altogether, these results show that COMET reproducibly measures protein abundances within the range where effects from altered protein abundance are anticipated to be detected.

### Identification of known ligase-substrate interactions

We next sought to test our ability to detect E3 perturbations that stabilize specific substrates. We separately examined data from HEK293 or K562 cells for differences in PSI distribution for each ORF paired with F-box-targeting gRNAs versus NTC gRNAs, which we term ΔPSI (PSI^targeting^ - PSI^NTC^; Figure S3C,D). After filtering for poorly represented gRNA-barcode or gRNA-ORF pairs, we identified 75 and 74 E3-substrate combinations whose PSI was significantly increased in HEK293 and K562 cells, respectively (*p* < 0.05; tests are two-sided t-test unless otherwise stated; *p*-values were corrected using the Benjamini-Hochberg method; Table S3).

**Figure 3.**
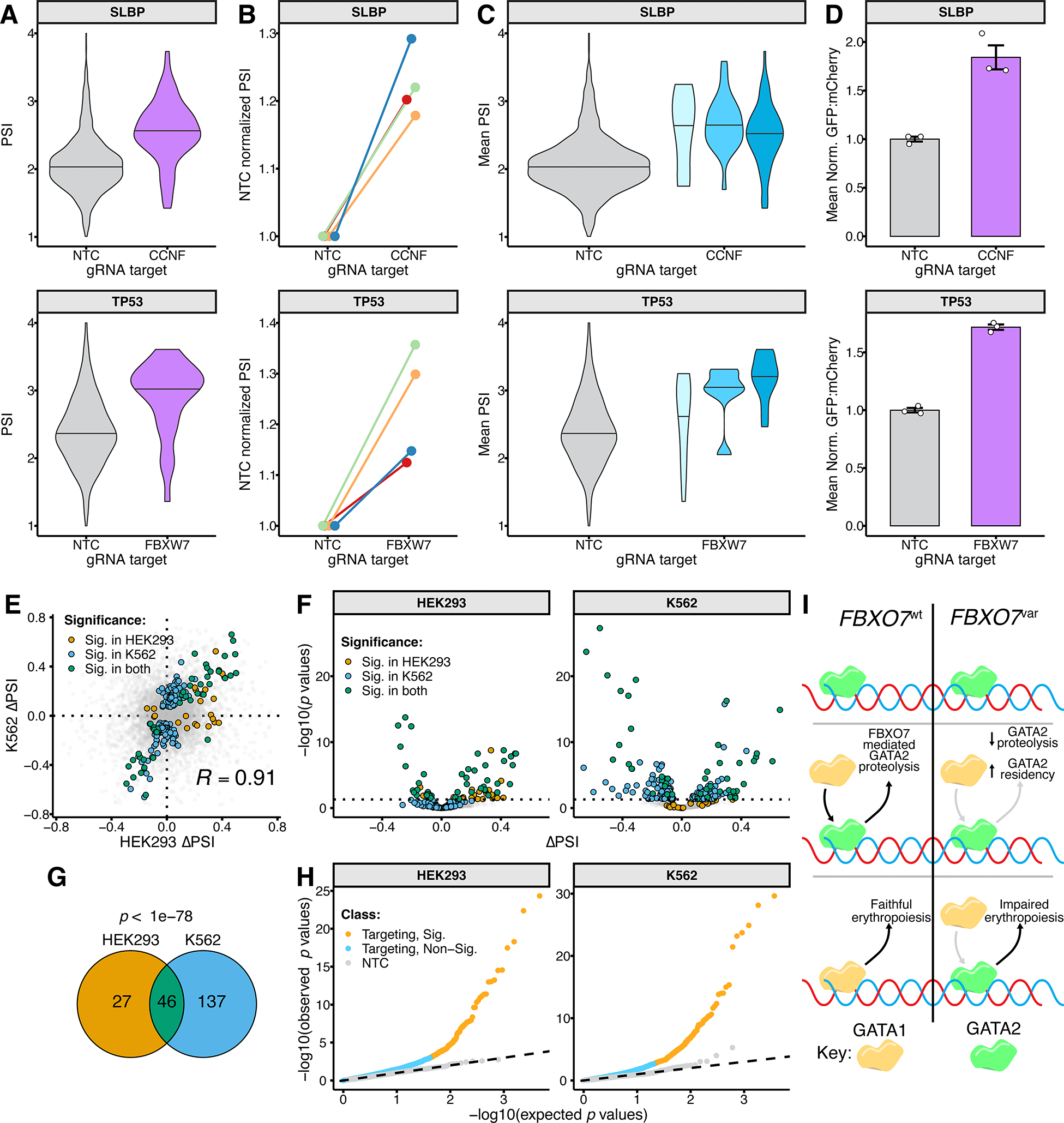
COMET identifies F-box substrate receptors for previously annotated SCF substrates. **A)** Violin plots of PSI for SLBP (top) and TP53 (bottom) in presence of NTC or targeting gRNAs for F-box genes CCNF (top) or FBXW7 (bottom). **B)** Line plots of NTC-normalized PSIs in each of four independent transfection replicates, as in panel **A** for CCNF-SLBP (top) and FBXW7-TP53 (bottom). **C)** Violin plots of mean PSIs in presence of NTC or three distinct gRNAs, as in panel **A** for CCNF-SLBP (top) and FBXW7-TP53 (bottom). **D)** Flow cytometry validation of CCNF-SLBP (top) and FBXW7-TP53 (bottom) effects. NTC-normalized mean GFP-mCherry ratios are shown. **E)** Comparison of ΔPSI estimates between HEK293 vs. K562. Pearson’s *R* calculated based on subset of interactions that are significant in either or both cell lines. **F)** Volcano plot of ΔPSI versus *p*-values in HEK293 (left) or K562 (right). Points colored based on whether the corresponding E3-substrate pair was significant in HEK293 (orange), K562 (blue), both (green) or neither (gray) cell line. **G)** Overlap of significant associations between HEK293 and K562 experiments. Significance of overlap calculated with hypergeometric test. **H)** Quantile-quantile plots from the HEK293 (left) or K562 (right) showing enrichment of measured *p*-values for both significant (yellow) and nonsignificant (blue) targeting gRNAs or NTC gRNAs (gray) over the null distribution of *p*-values (dashed line). **I)** Schematic of potential mechanism of GATA2→GATA1 switch regulation by FBXO7.

Focusing first on well-established E3-substrate pairs that we had included in the experiment, we successfully identified the founding F-box family member CCNF as controlling the abundance of SLBP^13^, in both HEK293 (ΔPSI = 0.23; *p* < 6e-6) and K562 (ΔPSI = 0.47; *p* < 6e-13) cells. Knockout of FBXW7 also increased the abundance of TP53 in K562 cells (ΔPSI = 0.50; *p* < 2e-3), a pairing that has been described under physiological conditions^14^. To visualize these effects, we plotted distributions of PSIs calculated at the barcode-guide level (Figure 3A). This confirms that the shifts in PSI distributions for SLBP and TP53, when paired with NTC versus CCNF- or FBXW7-targeting gRNAs, are not driven by outlier barcodes. Further, PSI increases for SLBP and TP53 were consistent across four transfection replicates, and across different targeting gRNAs (Figure 3B,C). Finally, we individually cloned SLBP and TP53 ORFs into the COMET reporter and integrated the construct in K562-Cas9-rtTA cells. Transfection of SLBP or TP53 reporter cells with gRNA plasmids targeting CCNF or FBXW7 resulted in a 1.8-fold and 1.7-fold increase in the mean GFP:mCherry ratio, respectively (Figure 3D).

### Reproducibility within and between cell lines

For each cell line, we arbitrarily collapsed reads from four independent transfection replicates to two replicates, and recalculated *p*-values and ΔPSIs. There were 57 (HEK293) and 126 (K562) E3-substrate pairs that were significant in one or both replicates. The ΔPSI values of pairs that were significant in both replicates were highly consistent (green points; Figure S4A,B; Pearson’s R = 0.97 and 0.98 for HEK293 and K562 respectively). Finally, considering all pairs that were significant in either replicate, we observe concordant direction of effects (HEK293: 55/ 57; *p* < 2e-14; K562: 124/126; *p* < 3e-16; binomial test).

We next examined the consistency of effects between cell lines. Across both HEK293 and K562 experiments, 209 unique E3-substrate pairs significantly shifted ΔPSI values either positively or negatively, with 46/209 pairs achieving significance in both cell lines (Figure 3E,F). Direction of effects were overwhelmingly concordant for shared significant associations (green points in Figure 3E; 45/46; *p* < 7e-13, binomial test; 19 destabilizing, 26 stabilizing). This directional concordance extended to pairs that were significant in only one cell line (orange [HEK293] and blue [K562] points in Figure 3E; 124/163; *p <* 1e-11, binomial test). Furthermore, the overlap in hits between cell lines was highly significant (*p* < 1e-78, hypergeometric test; Figure 3G). These patterns suggest that a larger proportion of the 209 pairs are shared, and might have been called as significant in both cell lines with greater experimental power.

In both HEK293 and K562, we observed a clear excess of significant *p*-values (Figure 3H), including numerous perturbation-ORF pairs discussed above that result in a significant PSI increase (HEK293: 75; K562: 74). Surprisingly however, we also observed many perturbation-ORF pairs that resulted in a significant PSI decrease (HEK293: 36; K562: 109). Assuming all effects are direct and E3 ligases are solely destabilizing, knocking out an E3 is only expected to increase the abundance of its target substrate, such that this is difficult to explain.

The majority of destabilizing pairs involved two ORFs, CTNNBIP1 (HEK293: 13; K562: 38) and SERINC3 (HEK293: 8; K562: 36). What might explain their recurrent destabilization? Multi-subunit E3s such as the SCF are dynamic complexes that exist in an equilibrium in which substrate receptors and substrates are constantly exchanged. It is possible that knockout of one substrate receptor disrupts this equilibrium leading to increased degradation of particular substrates. As our statistical tests detect differences between the means of the NTC and targeting gRNA PSI distributions, another possibility is that DNA damage caused by Cas9 double-strand breaks (DSBs) leads to a cellular environment in which CTNNBIP1 and SERINC3 are degraded.

We reasoned that if gain-in-activity of an untargeted substrate receptor underlies the recurrent destabilization of CTNNBIP1 and SERINC3, then these might share a common E3 knockout that stabilizes their abundance. Intriguingly, CTNNBIP1 and SERINC3 are stabilized by knockout of FBXW7 in K562 cells (as is SERINC3 in HEK293T cells). FBXW7 is a transcriptional target of the p53 TF^15^. p53 has a well-studied role as a regulator of the DNA-damage-repair pathway^16^, with p53 protein levels increasing in response to DNA damage. This suggests a mechanism in which the CRISPR-Cas9 DSBs leveraged by COMET to disrupt E3s leads to p53-mediated increases in FBXW7 levels and consequent degradation of the FBXW7 substrates CTNNBIP1 and SERINC3. On the other hand, we note that common SCF component knockouts (NEDD8, SKP1, RBX1) are three of the four most significant hits for these two ORFs, in the destabilizing direction. This supports an alternative hypothesis in which the SCF plays a direct or indirect role in stabilizing these two ORFs (and possibly other ORFs).

### Features of potential ligase-substrate interactions

Among the 75 significantly destabilizing F-box-ORF pairs in HEK293 cells, there were 26 unique ORFs, of which 16 (62%) are established SCF substrates. Considering only 30/92 ORFs in this experiment were previously SCF-linked, this represents a 1.9-fold enrichment (*p* < 6e-6, hypergeometric test). We observed a similar result in K562 cells (26 unique ORFs among 74 destabilizing pairs, 17 (65%) of which are established SCF substrates; 2.0-fold enrichment; *p* < 5e-6, hypergeometric test).

Although previously implicated as SCF substrates by GPS^7^, the F-box proteins responsible for their degradation were largely unknown since this information is not recoverable from a GPS experiment. Each of these ORFs was, on average, associated with multiple perturbations (mean 2.9 [HEK293] and 2.8 [K562]). ORFs associated with 3+ perturbations were dominated by previously annotated SCF substrates (HEK293: 11/14; K562: 9/9). Because previous GPS experiments utilized a dominant negative CUL1 fragment, our results suggest screens utilizing such fragments are biased towards recovering substrates surveilled by many individual F-boxes.

Degradation of these ORFs was mediated by 22 (HEK293) and 24 (K562) unique F-box or core SCF component proteins. Core SCF components RBX1 and SKP1, and the SCF regulator NEDD8, were recurrently implicated, often paired with substrates with additional F-box hits. For example, in K562 cells, we found CKS1B PSIs were increased when any one of the core SCF components (CUL1, SKP1, RBX1) or the CRL regulator, NEDD8, were knocked out, or an additional 7 F-box substrate receptors.

One might have predicted knocking out core complex components would have resulted in more substrate associations or perhaps a superset of associations. Of the 26 ORFs with at least one significant, stabilizing association in K562 cells, only 8 had at least one significant CUL1, SKP1, RBX1, or NEDD8 hit. However, this paucity could be due in part to insufficient power consequent to the highly essential functions of these core SCF components. This interpretation is supported by our observation that targeting of SKP1, RBX1, NEDD8, or CUL1 was underrepresented among gRNAs recovered from gDNA in both cell lines (Figure S4C,D,F,G). However, it is challenging to conclusively interpret a lack of an association because these potential false negatives may also be attributable to unknown factors, such as buffering effects of residual protein or subunit redundancy.

FBXW7 was the most recurrently observed F-box protein among K562 hits, paired with many cell cycle related proteins (*e.g.* CDK2AP1, CDK4, CDKN1A, CKS1B), consistent with the known role of FBXW7 as a tumor suppressor. In our data, FBXW7 knockout resulted in stabilization of GATA2, a key regulator of hematopoiesis^17^. While an association between FBXW7 and GATA2 has been previously reported^18^, our results suggest GATA2 may be degraded by two additional F-box proteins, FBXL16 and FBXO7. GATA2 auto-regulates its own expression during early hematopoiesis, eventually being displaced by GATA1 (“GATA switching”), leading to repression of *GATA2* expression and the installation of gene expression programs that drive erythropoiesis. GATA factor switching is driven at least partially by the drastic difference in half-lives for GATA1 (>4 hours) and GATA2 (~1 hour)^19^. Interestingly, a genome-wide association study associated *FBXO7* variants with red blood cell phenotypes, specifically an increase in mean cell hemoglobin^20^. Further, an *Fbxo7*-knockout mouse model exhibited impaired erythropoiesis and anemia with increased mean cell hemoglobin concentration^21^. We hypothesize that FBXO7-mediated GATA2 degradation enhances GATA switching by reducing GATA2 abundance and facilitating GATA1-driven erythropoiesis (Figure 3I, left). Reduction or loss of FBXO7 may impair the ability of GATA1 to displace GATA2, leading to impaired erythropoiesis (Figure 3I, right).

### Massively parallel testing for E3 ligases that surveil TFs with short half-lives

**Figure 4.**
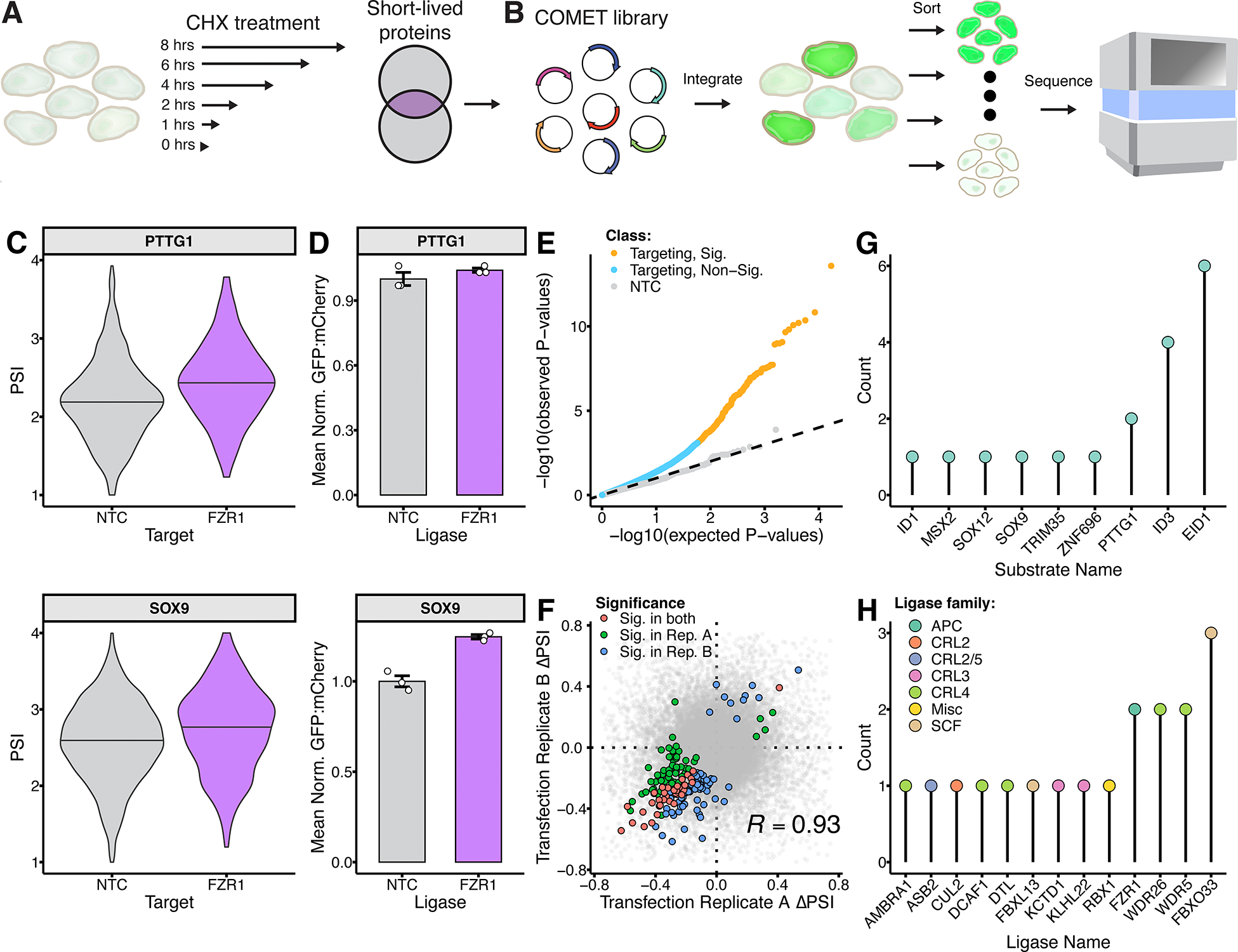
Screening for CRLs that degrade short-lived TFs. **A)** Schematic of experiment identifying short-lived TFs^28^. **B)** Schematic of application of COMET to these TFs. **C)** PSI distributions for PTTG1 (top) and SOX9 (bottom) when paired with NTC or *FZR1*-targeting gRNAs. **D)** Validation of *FZR1* knockout effects in PTTG1 and SOX9 reporter cell lines by flow cytometry. NTC-normalized mean GFP-mCherry ratios are shown. **E)** Quantile-quantile plot showing enrichment of measured *p*-values for targeting (yellow & blue points) or NTC gRNAs (gray points) over the null distribution of *p*-values (dashed line). **F)** Reproducibility of ΔPSI estimates. For this analysis, reads from two pairs of independent transfection replicates were combined to Replicates A & B, and ΔPSIs and significance was recalculated separately for each. Points colored based on whether corresponding E3-ORF pair was significant in Replicate A (blue), Replicate B (red) or both (green), or involves a NTC gRNA (gray). Pearson correlation calculated based on subset of interactions significant in Replicate A and/or Replicate B. **G)** Lollipop plot of 9 ORFs observed among 18 significant, positive interactions. **H)** Lollipop plot of 13 E3 perturbations observed among 18 significant, positive interactions.

Our results with GATA2 led us to wonder whether proteolytic degradation of short-lived TFs is a general phenomenon. Regulation of gene expression by TF proteolysis is well-established yet understudied^22–24^, with therapeutic implications as E3-TF interactions can be pharmacologically targeted for stabilization^25,26^ or abrogation^27^. Leveraging a recent study that identified short-lived proteins (half-life ≤ 8 hours) across a range of cell types^28^, we focused on 108 TFs and sought to ask whether their rapid turnover was attributable to proteasomal degradation, and if so, via which E3s (Figure 4A). Endogenous expression of these TFs in K562 cells was variable (Figure S2B,D).

While our initial experiment focused on perturbing F-boxes, we expanded the scope of our gRNA library to 723 gRNAs targeting 241 genes encoding components of the seven CRL families (SCF, CRL2, CRL3, CRL4A, CRL4B, CRL5, CRL7) and the anaphase-promoting complex (APC/C) ubiquitin ligase, supplemented with 50 NTC gRNAs. Altogether, we cloned a COMET library with 83,484 (773 gRNA x 108 ORFs) barcoded constructs and integrated these to K562-Cas9-rtTA cells (Figure 4B).

At 48 hours after reporter expression induction, cells were sorted based on the GFP:mCherry ratio, genomic DNA isolated from each of four FACS bins, and the gRNA-barcode region amplified and sequenced. Testing for significantly altered PSI distributions was performed as previously, revealing 9 TFs whose abundances were significantly increased by 13 ligase perturbations. Similar to the SCF-focused experiments, read proportions for gRNAs targeting CRL subunits were significantly different from all other gRNAs in the pool (Figure S4E,H). This phenomenon is less pronounced in the TF screen, which may be attributable to its expanded scale leading to less power to detect fitness effects and/or to the inclusion of more non-essential CRL subunits.

We identified PTTG1 and SOX9 as stabilized upon knockout of *FZR1*, a subunit of APC/C, a multisubunit protein assembly^29^ that controls cell cycle progression by degrading cell-cycle proteins such as cyclin B^30^ and PTTG1^31^ (Securin) (Figure 4C), supporting the former and suggesting the latter as APC/C substrates, results we validated in singleton experiments We validated these results in singleton experiments (Figure 4D). The APC/C is known to recognize its protein substrates through linear degradation motifs called degrons, including the D-box^32^ (RxxL) and KEN-box^33^ (KEN) motifs. There are two canonical APC/C coactivators responsible for APC/C substrate recognition, CDH1 (encoded by *FZR1*) and CDC20. Although PTTG1 was stabilized by knockout of *FZR1*, we did not detect a significant stabilization of PTTG1 following knockout of *CDC20* (ΔPSI = 0.15, *p* = 0.49).

The PTTG1-encoding ORF used in our screen has an N-terminal KEN-box and a more centrally located D-box; however the SOX9 ORF contains only a D-box. It is thought that degrons occur in disordered regions of substrate proteins^34^. However, the putative SOX9 D-box degron occurs in a highly structured alpha-helix within the DNA-binding domain. SOX9 is known to contain two nuclear-localization signals^35^ (NLS) and one nuclear-export signal^36^ (NES). Interestingly, the SOX9 D-box directly overlaps one of these NLS signals, suggesting APC/C may serve to not only degrade SOX9 but also to alter SOX9 localization.

A quantile-quantile plot again showed an excess of signal for targeting gRNAs (Figure 4E). As previously, upon recalculating ΔPSIs on independent pairs of replicates, we observe strong reproducibility for both the directionality and magnitude of significant ΔPSI values (Pearson’s R = 0.93; 200/207 directionally concordant; Figure 4F). Compared with our SCF-focused proof-of-concept, this scaled screen identified relatively few E3 perturbations that stabilize substrate abundance, with the PSIs of only 9 substrates increasing in response to 13 E3 perturbations (Figure 4G,H). This could result from having fewer test proteins with *a priori* evidence of being a E3 substrate, as well as reduced power due to this screen being more complex (83,484 rather than 22,264 barcoded constructs).

Once again, we also observed *destabilizing* effects, including 5 ORFs (ZNF593, CDKN2A, SLBP, TFAP4, TCEAL8) associated with >=25 destabilizing perturbations, and 7 ligases that, when knocked out, destabilized >=10 ORFs (DCAF7, ELOC, SKP2, WDR5, ASB16, SKP1, VHL). Recurrently destabilized ORFs may be sensitive to DSBs from Cas9 or some other general indirect interaction. Recurrently destabilizing perturbations may indicate a general role for the corresponding components in protein stabilization. For example, although WDR5 was included in our list of multi-subunit CRLs, it is better recognized as a core component of COMPASS complexes^37^, suggesting that COMPASS complexes may serve to stabilize certain TFs.

### Classifying the landscape of ligase-substrate interactions

We sought to ask whether any general patterns could be discerned in the distribution of E3-substrate interactions. For this analysis, associations involving core ligase components such as RBX1 or cullin scaffolds were excluded. Filtering further for significant, stabilizing associations (*p* < 0.05, ΔPSI > 0), COMET associated 48 E3s (SCF: 37, TF: 11) with 51 substrates (SCF: 42, TF: 9) for a total of 103 interactions across both experiments (SCF: 87, TF:16). We enumerated four classes of E3-substrate relationships: one-to-one, many-to-one, one-to-many, and many-to-many. For example, in our SCF HEK293 dataset, FBXW7 regulates 18 substrates including TP53, which itself is regulated by a single ligase, and so the interaction between FBXW7 and TP53 is categorized as many-to-one. With the caveat that we focused on only CRLs and did not test all possible substrates in our screens, we find examples of each class in each experiment (Table S4; Figure S5). Although we have not yet tested all E3s against all potential substrates, these results give us a preliminary sense for the connectivity of the proteolytic regulatory network.

### Computational assessment of COMET-nominated E3-substrate interactions

COMET nominates E3-substrate pairs for which one-by-one experimental validation (*e.g.* by co-immunoprecipitation) would be challenging to scale, and potentially to interpret due to technical confounders (*e.g.* transient E3-substrate interactions or substrate degradation). As a more scalable means of orthogonal assessment, we turned to *AlphaFold-Multimer*^38,39^, computing models for: 1) all 103 significantly stabilizing COMET-nominated E3-substrate pairs (class: ‘COMET-linked’); 2) As negative controls, 1,000 nonsignificant E3-substrate pairs randomly selected from the sets tested in our COMET experiments (class: ‘screen-negative’); 3) As additional negative controls, 1,000 pairs obtained by randomly sampling the canonical isoforms of all human proteins (class: ‘true-random’); 4) As positive controls, we extracted 1,000 E3-protein pairs from the BioPlex^40^ dataset (class: ‘BioPlex’), which is a proteome-scale map of experimentally derived protein-protein interactions generated via affinity-purification mass spectrometry.

Altogether, we modeled 3,103 protein-protein pairs with *AlphaFold-Multimer*, with 5 individual models per pair for a total of 15,515 unique models. To systematically evaluate support for a given interaction, we extracted interchain contacts from each model. Interchain contacts were defined based on the following criteria: 1) a maximum distance of 5 Å between any two atoms in the interacting residues; 2) a predicted aligned error (PAE) of ≥10 for each residue pair involved in the contact; and 3) a predicted local distance difference test (pLDDT) score of ≥50 for both residues. This strategy was adapted from a recent *AlphaFold-Multimer*-based *in silico* screen^41^.

**Figure 5:**
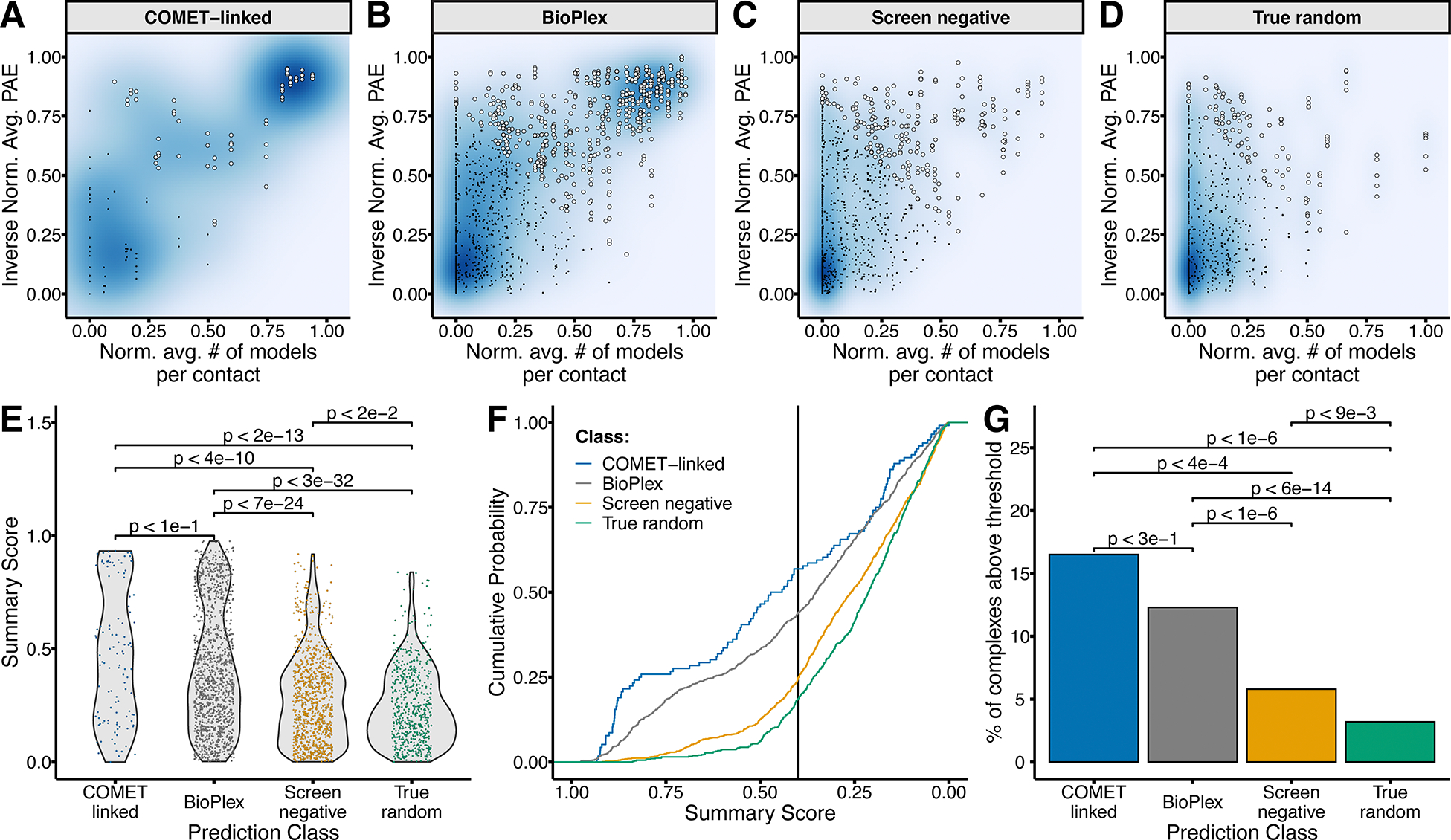
*AlphaFold-Multimer* modeling of COMET-nominated E3-substrate interactions **A)** Scatterplot of inverse normalized average PAE values plotted against the normalized average number of contacts per residue for the 103 COMET-nominated E3-substrate *AlphaFold-Multimer* predictions. Each point represents a single model. White points indicate models with a summary score >= 0.4. Black points represent models with a summary score <= 0.4. **B)** Same as in **A**, but for 1000 E3-protein pairs from BioPlex. **C)** Same as in **A**, but for 1000 nonsignificant E3-substrate pairs randomly sampled from those tested in COMET experiments. **D)** Same as in **A**, but for 1000 randomly sampled protein-protein pairs randomly sampled from the human proteome. **E)** Violin plot showing distribution of summary scores for COMET-nominated (blue), BioPlex (grey), screen-negative (orange) or true-random (green) prediction classes. Significance calculated using Wilcoxon rank sum test. **F)** Cumulative density function showing proportion of models exceeding range of summary score thresholds for each of the four prediction classes. Vertical black line indicates the summary score threshold (0.4) that we used. **G)** Barplot of proportion of complexes with ≥2 models passing the summary score threshold for each prediction class. Significance calculated using Fisher’s exact test, *p* values are adjusted with the Benjamini–Hochberg procedure.

Two metrics were calculated to summarize model quality: 1) *Inverse normalized PAE:* The mean PAE for all contacts of each individual model were calculated. These values were min-max normalized across all four prediction classes and inverted such that 0 corresponds to the lowest, and 1 to the highest, PAE values. 2) *Normalized contact consistency:* We summarized contact consistency as the average number of models each contact was observed in (range 1–5), similarly normalizing these values such that 0 corresponds to the lowest, and 1 to the highest, contact consistency. Plotting these metrics revealed a subset of models exhibiting high confidence and consistency in the COMET-linked and BioPlex classes but not the screen-negative or true-random classes (Figure 5A–D; note there are 10-fold fewer models in COMET-linked class than each control class). We also calculated a summary score which gave equal weights to confidence and consistency metrics, ranging from 0 (worst) to 1 (best). Encouragingly, summary score distributions for the COMET-linked and BioPlex classes were significantly higher than both negative control classes (Figure 5E).

We visualized a cumulative density function for each prediction class, and heuristically set a requirement that at least 2 of the 5 models for a given protein pair pass a threshold summary score of 0.4 to be considered supported (Figure 5F). 16.5% (17/103) of COMET-nominated pairs met these requirements, which is 1.3-fold higher than the BioPlex pairs (12.3%, 123/1000), 2.8-fold higher than the screen-negative pairs (5.8%, 58/1000) and 5.2-fold higher than the true-random pairs (3.2%, 32/1000). In summary, the odds of COMET-nominated pairs accruing *AlphaFold-Multimer* support was similar to E3-protein pairs from BioPlex (*p* < 3e-1, OR = 0.7, 95% CI = 0.4–1.3), and significantly greater than either screen-negative controls (*p* < 3e-4, odds ratio (OR) = 3.2, 95% CI = 1.7–5.9, Fisher’s exact test) or true-random controls (*p* < 5e-7, OR = 6.0, 95% CI = 3.0–11.6, Fisher’s exact test) (Figure 5G).

### Visualization of *in silico* models of E3-substrate interactions

**Figure 6.**
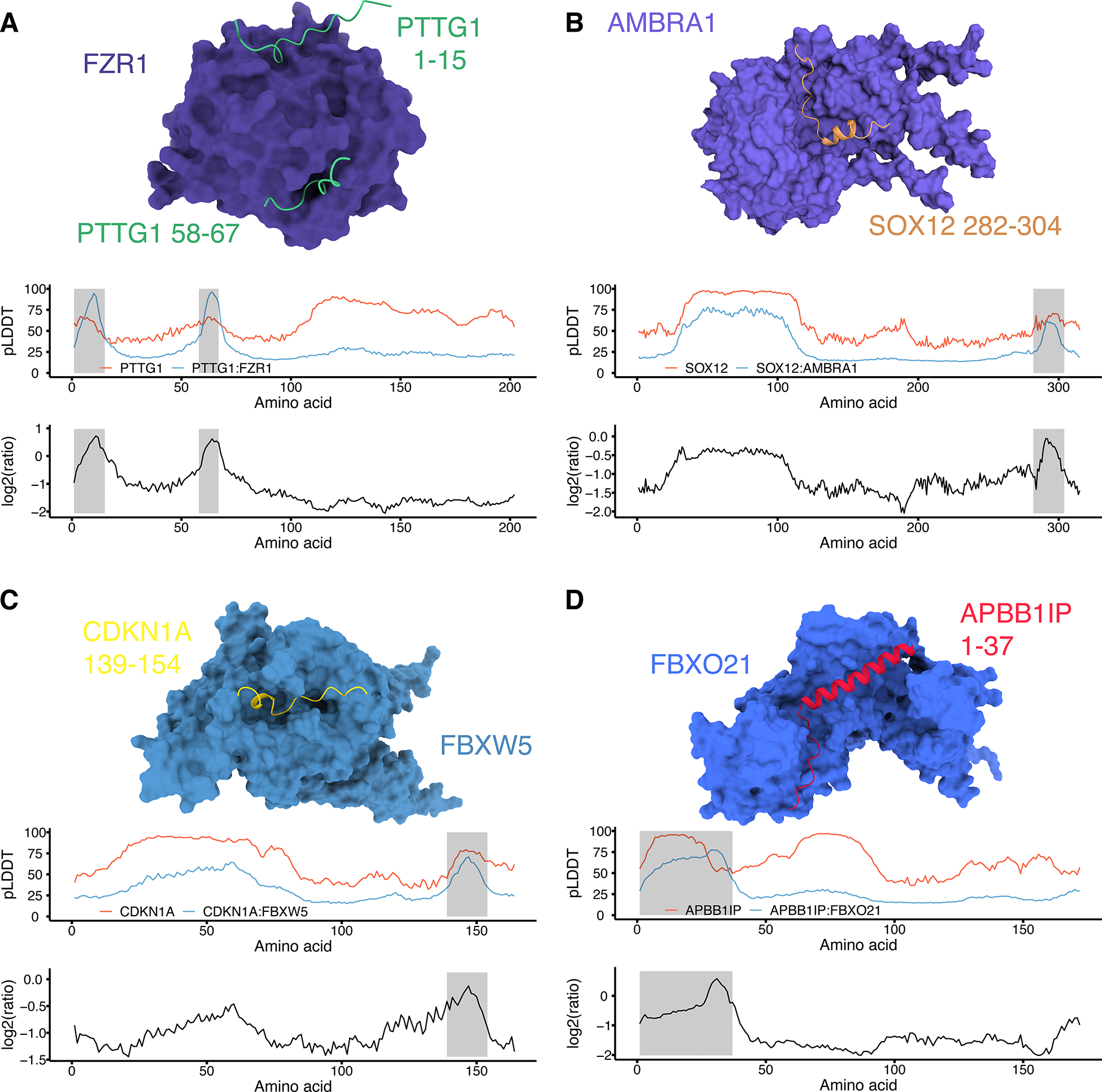
Computational models of E3-substrate interactions. **A-D)** (Top) Visualizations of FZR1-PTTG1 (**A**), AMBRA1-SOX12 (**B**), FBXW5-CDKN1A (**C**) and FBXO21-APBB1IP (**D**) modeling are presented. E3-substrate pairs were folded together with *AlphaFold-Multimer* and interacting substrate peptides highlighted. (Middle) Line plots of pLDDT values across the substrate protein are visualized in the context of a folded substrate monomer (red) or the substrate co-folded with the COMET-nominated E3 (blue). Finally, the log2-scaled ratio (monomer/complex) of pLDDT values at each residue are plotted, with peaks indicating regions of elevated pLDDT values in the complex prediction relative to the monomer. Note that scales for log2 ratios are different in each panel, *i.e.* adjusted for each plot to maximize contrast.

We next sought to examine selected *AlphaFold-Multimer*-derived models of COMET-nominated E3-substrate interactions in more detail, beginning with the well-established interaction of PTTG1 (Securin) and the APC/C adapter protein FZR1. As discussed above, FZR1 interacts with its substrates via degrons such as the KEN-box and D-box (RXXL) motifs. PTTG1, a known FZR1 substrate, contains KEN-box (residues 9–11) and more central D-box (RKAL, residues 61–64) motifs. To investigate this interaction further, we used *AlphaFold* to fold PTTG1 without FZR1 (Figure 6A; red curve). The resulting pLDDT values varied greatly across the protein, but were unremarkable near the KEN-box and D-box motifs. However, when we fold PTTG1 with FZR1 via *AlphaFold-Multimer*, we observe two striking increases in local pLDDT values that precisely coincide with the degrons (Figure 6A; blue curve). Furthermore, computational substrate-E3 co-folding induced a reduction of pLDDT values in all regions outside of these degrons. This result suggests the potential for co-folding of substrates with their cognate E3s to result in (relatively) increased model confidence localized to degron motifs, *i.e.* presumably corresponding to E3 engagement with the degron(s).

We also examined three COMET-nominated E3-substrate interactions that are previously undescribed, supported by structural modeling, and exhibit coincident patterns with respect to co-folding confidence and putative degron locations. In the first of these, we folded SOX12 with vs. without its COMET-associated E3, AMBRA1. Once again, we observe a relative jump in the pLDDT complex/monomer ratio, in this case a narrow C-terminal region, suggesting this region may correspond to a degron (Figure 6B). SOX12 and AMBRA1 have not been shown previously to interact. However, AMBRA1 was recently shown to recognize its various Cyclin D substrates through a conserved TP motif^42^ and the *AlphaFold-Multimer* model contains a TP motif at the center of the putative degron (Figure S6E,F). Interestingly, this putative degron (residues 282–304) also overlaps the majority of the SOX12 transcriptional activation domain (residues 283–315), consistent with the observation that degron motifs within TFs often overlap the transcriptional activation domain^24^.

The third example involves the cyclin-dependent kinase inhibitor CDKN1A and F-box protein FBXW5 (Figure 6C). Here, the CDKN1A peptide predicted to be bound by FBXW5 was confidently predicted even when CDKN1A is modeled on its own (Figure 6C). Although the pLDDT values around the predicted interacting region are not increased as in the previous two examples, the range of high-confidence pLDDT values is narrowed upon co-folding. As previously mentioned, CDKN1A functions as a cyclin-dependent kinase inhibitor. Cyclins interact with their binding partners through Cy motifs (*e.g.* RxL or RxI) on the partner protein. CDKN1A contains one N-terminal (RRL, residues 19–21) and one C-terminal (RRL, residues 155–157) Cy motif. The C-terminal Cy motif is just outside of the predicted interacting region, raising the possibility that FBXW5 may sequester CDKN1A or otherwise compete for binding with other CDKN1A interactors. Beyond our experimental and computational association of FBXW5 to the cell cycle related CDKN1A, FBXW5 has previously been shown to degrade other cell cycle proteins such as the centriole assembly factor SAS-6^43^ and the actin regulator EPS8^44^.

The fourth example involves APBB1IP and FBXO21. Similar to the CDKN1A-FBXW5 interaction, the putative APBB1IP degron (residues 139–154) exhibited consistently high pLDDT values in the monomer prediction (Figure 6D). However, the pLDDT values of the predicted interacting residues decreased following upon co-folding with FBXO21. Of note, the putative APBB1IP degron is the only example presented here that is structured; this suggests co-folding partially disrupts the predicted secondary structure of this region leading to the observed decrease in pLDDT. A previous study demonstrated that FBXO21 is responsible for degrading the short-lived protein EID1^45^ and mapped the minimal EID1 degron to residues 160–172. Alignment of APBB1IP residues 1–37 notably revealed two APBB1IP residues (F12 and L16) were conserved with EID1 and its paralog, EID2, as well as two semi-conserved, hydrophobic residues (L21 and L22) (Figure S6G,H). Plots visualizing PAE values for each of the above examples are provided in Figure S6A–D.

To test whether *AlphaFold-Multimer*-nominated degrons mediate degradation in an E3-specific manner, we cloned a series of reporters expressing full-length substrate proteins or their putative degrons fused to GFP, integrated these into HEK293-rtTA-Cas9 cells together with a construct expressing either an E3-specific or NTC gRNA, and measured GFP:mCherry ratios 14 days post-transfection. Cells co-transfected with constructs bearing an N-terminal GFP fusion of PTTG1 and an FZR1-targeting gRNA exhibited a 1.4-fold increase in their GFP:mCherry ratio relative to controls (Figure S7A; *p* = 0.009, one-sided t-test). This ratio was similar when only the degron region of PTTG1 (residues 1–84) was fused to GFP (1.3-fold increase; *p* = 0.018). Splitting the degron into its KEN-box (residues 1–42) or D-box (residues 42–84) components resulted in no or modest (1.1-fold) stabilization, respectively, suggesting both motifs are required. Cells expressing a C-terminal GFP fusion of SOX12 and an AMBRA1-targeting gRNA exhibited 1.2-fold stabilization relative to controls (*p* = 0.018) while the putative degron region of SOX12 (residues 282–304) did not (0.9-fold of original) (Figure S7B). Cells transfected with C-terminal GFP fusions of full length CDKN1A or the CDKN1A degron (residues 130–164) showed no or modest (1.1-fold increase; *p* = 0.027) stabilization, respectively (Figure S7C), in the presence of a FBXW5-targeting gRNA. Finally, we tested N-terminal GFP fusions of full-length APBB1IP or the putative APBB1IP degron (residues 1–40), which strikingly exhibited 1.5-fold (*p* < 5e-6) and 1.8-fold (*p* = 0.003) stabilization, respectively, in the presence of a FBXO21-targeting gRNA (Figure S7D). Although further investigation into the mechanisms by which these putative degrons mediate substrate proteolysis is warranted, the observation of E3-dependent, degron-mediated proteolysis in several of the cases tested (FZR1-PTTG1, FBXW5-CDKN1A, FBXO21-APBB1IP) supports the utility of leveraging *AlphaFold-Multimer* as a means of degron discovery.

## Discussion

Protein degradation is an essential component of cellular regulation, but relatively few relationships between specific E3s and specific target substrates are known. Towards addressing this, we developed COMET, a combinatorial experimental framework wherein the consequences of perturbing many E3 ubiquitin ligases on the stability of each of many overexpressed candidate substrates can be tested in a single experiment. COMET is highly scalable, with tens-of-thousands of potential E3-substrate pairs assayable in a single experiment. In contrast with GPS screens which rely on dominant-negative fragments or pharmacologic inhibition, the CRISPR perturbations leveraged by COMET can be directed at non-CRL, monomeric ubiquitin ligases, *e.g.* RING, HECT, and RBR E3s^46^. Moreover, although unexplored in this study, the potential exists to use CRISPR activation of E3s in COMET screens.

**Figure 7.**
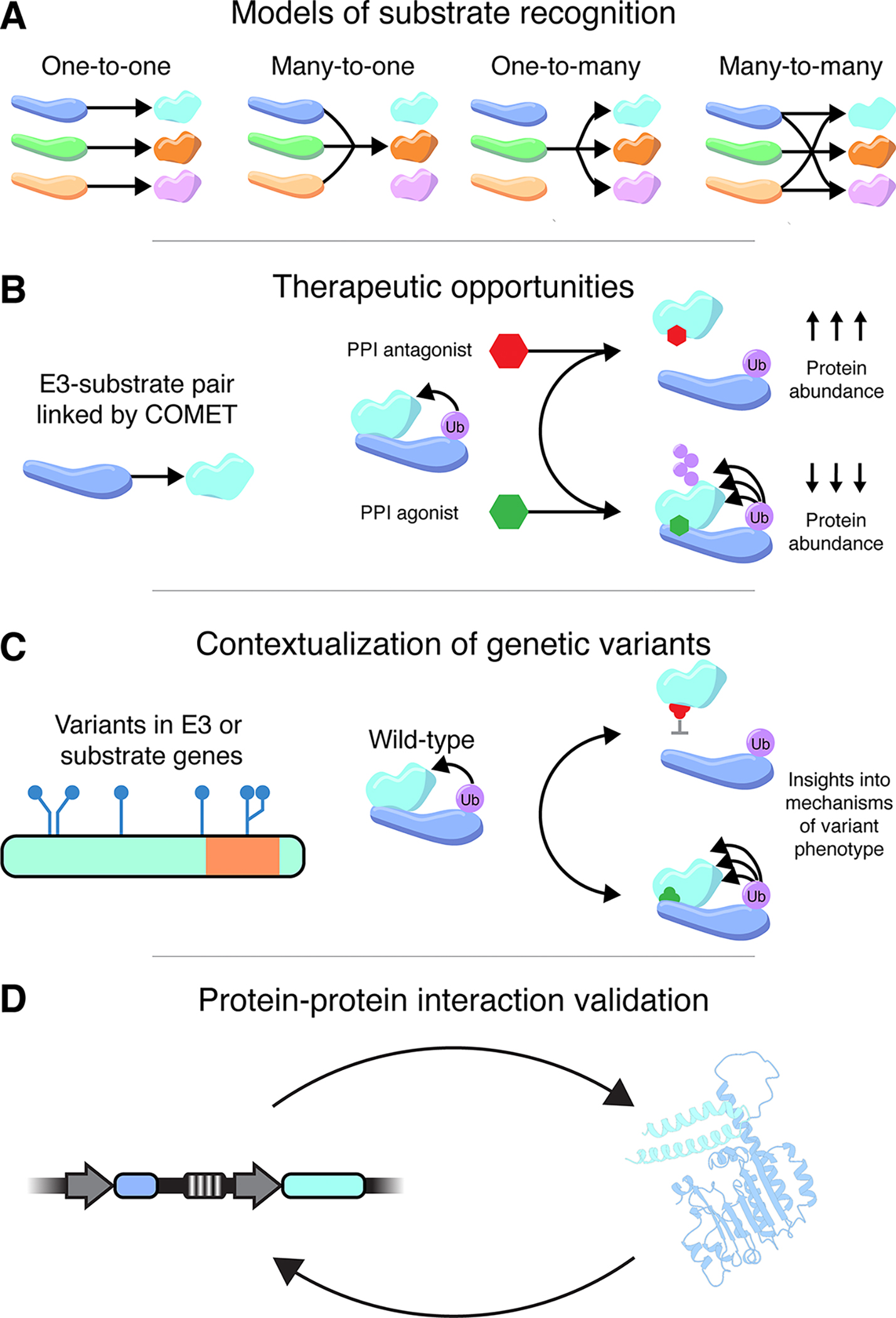
Potential applications of COMET. **A)** By linking E3s to their substrates, COMET advances our understanding of the connectivity of regulatory networks controlling protein abundance. Visualized are four possible classifications of E3-substrate relationships based on the number of interactions. **B)** Individual E3-substrate interactions represent potential therapeutic targets for either stabilization or inhibition. **C)** E3-substrate pairs linked via COMET may provide insights into the mechanisms by which E3 or substrate variants lead to disease phenotypes. **D)** Reciprocal validation of COMET or *AlphaFold-Multimer*-nominated E3-substrate interactions.

By expanding the number of experimentally supported E3-substrate interactions, we anticipate that COMET may advance models of proteolytic regulatory networks (Figure 7A). Such links may also be valuable for targeted protein degradation, *e.g.* via proteolysis-targeting chimeras (PROTACs) or molecular glues (MGs), which hold great therapeutic promise but are hindered by the dearth of endogenous E3-substrate interactions, *e.g.* to target for stabilization with MGs (Figure 7B). COMET may also be useful for contextualizing genetic variants in E3s and/or substrates (Figure 7C). As one example, dissecting the GWAS association of FBXO7 with red blood cell phenotypes is daunting given that the phenotype could arise from FBXO7 degrading nearly any substrate protein. However, the COMET-derived linkage of FBXO7 and GATA2 suggests a plausible mechanism for the GWAS association (Figure 3I). Of note, a recent analysis of 33 cancer types showed 19% of mutated cancer driver genes affect protein degradation^47^.

Although in principle, COMET makes it possible to test all possible E3s vs. all possible substrates, library construction and FACS would undoubtedly become bottlenecks, as testing 241 CRLs vs. ~20,000 human genes would require an experiment 185-fold larger than what we have demonstrated here. However, this challenge might be eased by upfront ORFeome-wide screens to identify bonafide proteasome or CRL substrates to be tested by COMET. For example, 1,554 proteins were recently shown to be stabilized by the pan-CRL inhibitor MLN4924, suggesting they are CRL substrates^48^. The comprehensive application of COMET to these substrates (241 CRLs x 1554 CRL substrates) would require an experiment only 14-fold larger than the short-lived TF COMET screen reported here, a challenging but approachable scale.

An alternative would be to invert the current framework (experimental nomination→computational validation) by instead first identifying candidate E3-substrate pairs through scaling of deep learning-based modeling to all possible interactions (computational nomination→experimental validation; Figure 7D). In this scenario, the computational resource requirements would be dramatically larger, but given the sparseness of the set of true interactions, experimental validation could likely be achieved in a single COMET experiment. A limitation is that the false negative rate of computational modeling of E3-substrate interactions is not known, but the same could be said of experimental screening methods.

From a resources perspective, what would this take? Here we generated *AlphaFold-Multimer* predictions for 3,103 E3-substrate pairs (*i.e.* 103 COMET-linked E3-substrate pairs and 3,000 control pairs). Each prediction required ~1 GPU-hour (mean: 1.8 GPU-hr; median: 1 GPU-hr), with total compute time of ~157 GPU-days. Although the tradeoff between prediction quality and speed could be adjusted, assuming identical parameters as used here (5 models, 30 recycles, model similarity tolerance of 0.5), we estimate modeling interactions between the ~375,000 possible pairs of 241 CRLs and 1554 CRL substrates would require ~43 GPU-years, which is substantial but certainly not impossible. Expanding this to all ~600 human E3s vs. all ~20,000 human proteins (~12 million pairs) would be more challenging (~1369 GPU-years), but for perspective we note that there are already over 200 million predictions in the AlphaFold Protein Structure Database (June 2024). Furthermore, predictions reported here were generated using a combination of Nvidia A100 and L40 GPUs; simply switching to faster GPU architectures would likely result in shorter prediction times.

Here we described COMET, a combinatorial framework for testing the role of many specific E3s in degrading many specific substrates within a single experiment, and furthermore demonstrated the potential for targeted computational modeling to provide orthogonal support for experimentally nominated E3-substrate pairs. However, given the pace at which structural modeling is improving and computational resources are scaling, we envision a future in which this framework is inverted, and methods like COMET are used for the multiplex experimental validation of predictions generated by computational modeling of a vast number of potential interactions (Figure 7D).

## Limitations of the Study

As COMET is a pooled assay, measured abundances are relative to other proteins in the pool, which makes it challenging to compare effect sizes between different experiments. This could potentially be mitigated by including protein spike-ins with known abundances to facilitate batch correction and/or by expanding the number of FACS bins.

Although our detection of some established E3-substrate pairs shows COMET can detect effects from directly interacting partners, it is possible that COMET-nominated E3-substrate pairs have an indirect relationship, *e.g.* if a targeted E3 regulates an untargeted E3 which then regulates a targeted substrate. This could potentially be mitigated by computational support for a given interaction, or by scaling the screen to target all E3s and deubiquitinases.

Finally, as with any large-scale screen, there exists the potential for both false negatives and false positives. False negatives could arise from insufficient power, ineffective gRNAs, essentiality of perturbed genes, substrates that lack the correct post-translational modifications necessary for ubiquitination, lack of E3 expression, or masking of degrons near the C-terminus by the EGFP fusion. False positives could arise from non-specific effects, *e.g.* off-target events or cellular responses induced by Cas9-induced DSBs^49^.

## RESOURCE AVAILABILITY

### Lead Contact

Further information and requests for resources and reagents should be directed to and will be fulfilled by the lead contact, Jay Shendure (shendure@uw.edu).

### Materials availability

All unique reagents generated in this study are available from the lead contact with a completed Materials Transfer Agreement.

### Data and Code Availability

- All sequencing data are deposited at GEO (GSE234621) and are publicly available. This paper analyzes existing, publicly available data, with accession numbers listed in the key resources table.
- All AlphaFold predictions are available at https://krishna.gs.washington.edu/content/members/COMET/public/ • This paper does not report original code.
- Any additional information required to reanalyze the data reported in this paper is available from the lead contact upon request.

## STAR★METHODS

### EXPERIMENTAL MODEL AND STUDY PARTICIPANT DETAILS

#### Cell lines and cell culture

K562 (CCL-243) and HEK293 (CRL-1573) cell lines were purchased from ATCC. K562 cells were cultured in RPMI 1640 (Gibco). HEK293 cells were cultured in DMEM, high glucose (Gibco). All media were supplemented with 10% FBS (Hyclone) and 1% penicillin-streptomycin (Gibco).

### METHOD DETAILS

#### Monoclonal cell line generation

K562 cells or HEK293 cells were transduced with lentiCas9-Blast (Addgene #52962-LV) lentivirus. Beginning 2 days post-transduction, transduced cells were selected with 10 μg mL^−1^ Blasticidin S HCl (Gibco, A1113903) for 6 days. The reverse tetracycline transactivator was then stably integrated into each polyclonal cell line via piggybac transposition. Polyclonal K562-Cas9 or HEK293-Cas9 were then transfected with 1500 ng of pB-rtTA (Addgene #126034) and 250 ng of transposase expression construct to stably integrate the reverse tetracycline transactivator. For transfection of K562 cells, 1 × 10^6^ K562 cells were nucleofected using a Lonza 4D-Nucleofector. For transfection of HEK293 cells, 1 × 10^6^ HEK293 cells were seeded in individual wells of a 6 well plate; cells were transfected 1 day post-seeding using Lipofectamine 3000 (Thermo Fisher). Beginning 2 days post-transfection, polyclonal K652-Cas9-rtTA or HEK293-Cas9-rtTA cell lines were selected with 5 μg mL^−1^ Blasticidin S HCl and 800 μg mL^−1^ G418 Sulfate (Gibco) for 6 days.

Monoclonal K652-Cas9-rtTA and HEK293-Cas9-rtTA cell lines were generated by limiting dilution of selected polyclonal cell populations. For each polyclonal cell line, cells were diluted to 5 cells ml^−1^ in non-selective media without selective antibiotics and 100 μL of diluted cells were added to individual wells of a 96 well plate and expanded for 14 days. Candidate monoclonal lines were screened for reporter induction and Cas9 activity via transfection of a GFP-IRES-mCherry reporter with either a NTC- or GFP-targeting gRNA (Figure S1).

##### Plasmid library cloning

###### Iterative library cloning strategy

COMET plasmid libraries were cloned in a four-step process (Figure S1).

In the first step, libraries of gRNA spacer sequences are cloned into the COMET backbone. To generate the backbone, pCCS_30 was digested with BsmBI-V2 (NEB) for 6 hours and purified by agarose gel purification (Monarch DNA Gel Extraction Kit, NEB). Spacer sequences targeting E3 ubiquitin ligases of interest were taken from the Brunello CRISPR knockout library^50^ and ordered as either oPools (IDT) or Oligo Pools (Twist Bioscience). Single stranded oligonucleotide libraries were diluted to 1 ng/uL and PCR amplified in a reaction containing 100 μL KAPA HiFi master mix (Roche), 1 μL 100 μM oCCS_90, 1 μL 100 μM oCCS_91, 2 μL 100X SYBR Green (Thermo Fisher), 8 μL 1 ng μL^−1^ oligonucleotide library, and nuclease-free water to 200 μL. Libraries were amplified with cycling parameters of 3 minutes at 95 °C followed by 12 cycles of 20 seconds at 98 °C, 15 seconds at 63.5 °C and 15 seconds at 72 °C. PCR products were cleaned up with a DNA Clean and Concentrator kit (Zymo) and subsequently Gibson assembled (NEBuilder HiFi DNA Assembly, NEB) into BsmBI-digested pCCS_30 using a 5:1 insert:backbone molar ratio at 50 °C for 30 minutes. Gibson assembly products were then cleaned up with a Clean and Concentrator kit, eluted in 6 μL water, and electroporated into ccdB Survival *Escherichia coli* (Thermo Fisher). Electrocompetent ccdB Survival cells were prepared in-house as an electrocompetent version of this strain is not commercially available. 1% of the electroporation was serially diluted and plated onto LB agar plates with ampicillin to quantify the number of transformants and ensure adequate library complexity. The remaining volume of electroporation was added to 100 mL of liquid LB media supplemented with 100 μg mL^−1^ ampicillin and cultured at 37 °C for 16 hours. Library plasmid DNA was then isolated (ZymoPURE II Plasmid Midiprep Kit) and used as input for the next round of cloning.

The second cloning step introduces libraries of open reading frames (ORFs) into the COMET library via Gateway cloning (Thermo Fisher). Gateway entry clones encoding ORFs of interest were obtained from DNASU. A Gateway reaction consisting of 28 μL 20 ng μL^−1^ Gateway entry clones, 4 μL 150 ng μL^−1^ COMET library DNA, 8 μL Gateway LR clonase (Thermo Fisher), and water to 40 μL was incubated at 25 °C for 16 hours. The reaction was stopped by addition of 2 μL Proteinase K (Thermo Fisher) to the reaction and incubating at 37 °C for 10 minutes, after which the library was cleaned up with a Clean and Concentrator kit, eluted in 6 μL water, electroporated into *Escherichia coli* (NEB, C3020), cultured at 37 °C for 16 hours, and plasmid DNA was then extracted. As before, 1% of the electroporation was serially diluted to enumerate transformants.

Next, the library was barcoded with a 25-mer degenerate barcode. The COMET backbone containing gRNAs and ORFs was digested in a reaction containing 12 μL I-SceI (NEB), 12 μL I-CeuI (NEB), 4 μL rAlbumin (NEB), 20 μL 10X CutSmart Buffer (NEB), 20 μg of vector, and water to 200 μL, incubated at 37 °C for 6 hours and purified by agarose gel purification. A DNA fragment containing a 25-mer random barcode was generated by mixing 1 μL 100 μM oCCS_440, 1 μL 100 μM oCCS_441, 4 μL NEBuffer 2.1, and water to 40 uL. To anneal oligos this mix was heated to 95 °C for 3 minutes and then cooled to 22 °C at a ramp rate of 0.1 °C per second. Annealed oligos were extended by adding 1 μL Klenow (NEB M0210S) and 1 μL 10 mM dNTPs (NEB) followed by a 20 minute incubation at 25 °C, after which the barcoded DNA fragment was cleaned with a Clean and Concentrator kit and Gibson assembled at 50 °C for 30 minutes using a 5:1 insert:backbone molar ratio. The Gibson product was cleaned up with a Clean and Concentrator kit, eluted in 6 μL water, and electroporated into *Escherichia coli* (NEB, C3020) which were cultured at 37 °C for 16 hours. 1% of the electroporation was serially diluted to enumerate transformants. Plasmid DNA was extracted and used as input for the final round of cloning. This plasmid DNA was also used for barcode subassembly, details of which are described in the *Subassembly of ORF-barcode pairs* section below.

The final step of the COMET cloning workflow introduces a tetracycline response element (TRE) promoter into the library. The COMET backbone containing gRNAs, ORFs, and barcodes was digested for 6 hours at 37 °C in a reaction containing 15 μL I-SceI (NEB), 12 μL 10X CutSmart Buffer (NEB), 12 μg of vector, and water to 120 μL and purified by agarose gel purification. An amplicon containing the TRE promoter was amplified from pCCS_11 in a reaction containing 25 μL KAPA HiFi master mix (Roche), 1 μL 10 μM oCCS_456, 1 μL 10 μM oCCS_457, 5 μL 10X SYBR Green, and 1 μL 1 ng μL^−1^ pCCS_11, and nuclease-free water to 50 μL with cycling parameters of 3 minutes at 95 °C followed by 16 cycles of 20 seconds at 98 °C, 15 seconds at 70 °C and 60 seconds at 72 °C. The PCR product was cleaned up with a Clean and Concentrator kit, Gibson assembled at 50 °C for 30 minutes using a 5:1 insert:backbone molar ratio, cleaned up with a Clean and Concentrator kit, eluted in 6 μL water, and electroporated into *Escherichia coli* (NEB, C3020) which were cultured at 37 °C for 16 hours. 1% of the electroporation was serially diluted to enumerate transformants. Plasmid DNA was extracted and used for stable integration into cells.

###### Subassembly of ORF-barcode pairs

Plasmid libraries with gRNAs, barcodes, and ORFs were tagmented with N5-loaded Tn5 transposase (Diagenode) in a reaction containing 100 μL 2x tagmentation buffer (Diagenode), 80 μL 2.5 ng μL^−1^ plasmid DNA, 3 μL 1.25 μM N5-loaded Tn5 transposase, and water to 200 μL with incubation conditions of 55 °C for 5 minutes and 10 °C for 10 minutes. Tagmented plasmid DNA was cleaned up with a Clean and Concentrator kit, eluted in 54 uL of water which was input into a PCR reaction containing a primer (oCCS_473) that hybridizes upstream of the barcode region and a primer (for example oCCS_P5_1) that hybridizes the tagmentation event and appends an Illumina sequencing index as well as the P5 sequencing adapter. The reaction contained 54 uL tagmented DNA, 850 μL NEBNext High-Fidelity 2X PCR Master Mix (NEB, M0541S), 85 μL 10 μM oCCS_473, 85 μL 10 uM P5 indexing primer (for example oCCS_P5_1), 170 μL 10X SYBR Green, and water to 1700 μL. This master mix was distributed into 34 wells of a 96 well PCR plate with 50 μL per well and amplified with cycling parameters of 5 minutes at 72 °C, 30 seconds at 98 °C followed by 10 cycles of 10 seconds at 98 °C, 30 seconds at 68 °C and 60 seconds at 72 °C. The PCR product was cleaned up using 0.8X AMPure XP Beads (Beckman Coulter), eluted in 100 μL of Buffer EB (Qiagen), and Illumina sequencing indexes were added in a second PCR reaction consisting of 85 μL 1st round PCR product, 850 μL of 2x NEBNext master mix, 85 μL 10 μM of the same P5 indexing primer from round 1 (for example oCCS_P5_1), 85 μL of P7 indexing primer (for example oCCS_P7_1), 170 μL of 10X SYBR Green, and water to 1700 μL with cycling parameters of 30 seconds at 98 °C followed by 10 cycles of 10 seconds at 98 °C, 30 seconds at 63 °C and 60 seconds at 72 °C. The PCR product was cleaned up using 0.8X AMPure XP Beads, eluted in 100 μL of Buffer EB. 20 uL of eluted product were run on a pre-cast 6% TBE polyacrylamide gel (Thermo Fisher), and DNA fragments ranging in size from 400–1000 base pairs were excised from the gel and purified. The resulting library was quantified and sequenced on an Illumina instrument.

###### Selection short-lived transcription factors

Transcription factors with short half-lives were identified from a proteome-wide map of short-lived proteins^28^. Briefly, this study applied multiplex mass spectrometry to four widely used human cell lines (HEK293T, U2OS, RPE1, HCT116) treated with the translation-inhibiting drug cyclohexamide (CHX) (Figure 4A). Using this approach, 1,017 proteins were identified as short-lived (half-life ≤ 8 hours) in at least one of the four cell lines tested. From this list of 1,017 short lived proteins, we retrieved all proteins that have evidence of being a transcription factor. This resulted in a set of 108 TFs that we then tested in the short-lived transcription factor COMET screen,

##### Screens

###### Library transposition and cell culture

COMET libraries were integrated into the genome of monoclonal K562-rtTA-Cas9 or monoclonal HEK293-rtTA-Cas9 cells by piggyBac transposition. In each experiment we conducted four biological replicates in parallel. For each replicate on day 0, 20 × 10^6^ cells were electroporated with 10 μg COMET library and 6 μg of transposase expression construct on a MaxCyte STX instrument using cell line specific (i.e. K562 or HEK293) electroporation programs. On day 2 cells were selected in media containing 2 μg mL^−1^ puromycin (Gibco) to select for cells containing COMET library plasmids. Cells were passaged such that 500X coverage of the library was maintained. Expression of COMET reporter constructs was induced on day 10 via addition of 1 ug mL^−1^ doxycycline to the media and cells were sorted on day 12.

###### Flow cytometry

Cells were sorted on a BD FACSAria II or a BD FACSymphony S6 (Becton Dickinson). Cells were gated from all events followed by gating on single cells. From the population of single cells, mCherry positive cells were gated, and a GFP:mCherry ratio parameter was created and applied to this population. A histogram of GFP:mCherry ratio was created and gates were drawn that divided the histogram into four equally partitioned bins containing approximately 24% of the gated population. Cells were sorted into four bins based on the GFP:mCherry ratio (low to high GFP:mCherry), and 100X coverage of the library complexity was sorted per biological replicate in each experiment. Sorted cells from each of the four bins were centrifuged and stored as pellets.

###### Generation of gRNA-barcode Illumina amplicons

Genomic DNA (gDNA) was extracted from pellets of frozen cells using a DNEasy Blood and Tissue kit (Qiagen). Illumina amplicons containing gRNA and barcode information were amplified from gDNA using two rounds of PCR. In the first round of PCR, an amplicon containing DNA barcodes and gRNA spacer sequences was amplified from the gDNA. All genomic DNA isolated from cells from each FACS bin was divided into 24 individual 50 uL PCR reactions consisting of 25 μL KAPA2G Robust HotStart ReadyMix (Roche), 0.25 μL 100 μM oCCS_495, 0.25 μL 100 μM oCCS_496, 0.5 μL 100X SYBR Green, 8 μL gDNA, and 16 μL of water with cycling parameters of 3 minutes at 95 °C followed by 23 cycles of 15 seconds at 95 °C, 15 seconds at 59 °C and 60 seconds at 72 °C. The PCR product was cleaned up using 1X AMPure XP Beads and eluted in 20 μL of Buffer EB per well of which 2.5 uL was used as input for a second round of PCR. The second round of PCR appended Illumina sequencing adapters and consisted of 2.5 μL of 1st round PCR product, 2.5 μL 10 μM P5 indexing primer (for example oCCS_P5_1), 2.5 μL 10 μM P7 indexing primer (for example oCCS_P7_1), 25 μL 2x NEBNext master mix, 0.5 μL 100X SYBR Green, and water to 50 μL with cycling parameters of 30 seconds at 98 °C followed by 5 cycles of 10 seconds at 98 °C, 30 seconds at 63 °C and 15 seconds at 72 °C. The PCR product was cleaned up using 1X AMPure XP Beads, eluted in 18 μL of Buffer EB. 5 uL of eluted product from each sample was pooled into a single sample and sequenced on an Illumina instrument.

###### Individual hit validation

Entry clones encoding putative substrates of interest were recombined via a Gateway LR reaction into pCCS_82. A Gateway reaction consisting of 7 μL 20 ng μL^−1^ entry clone, 1 μL 150 ng μL^−1^ pCCS_82, 2 μL LR Clonase II (Thermo Fisher) was incubated for 1 hour at 25 °C, terminated by adding 2 μL Proteinase K (Thermo Fisher) and incubating at 37 °C for 1 minutes, and transformed into *Escherichia coli* (Stellar cells, Takara, 636763). gRNAs targeting E3s of interest were PCR amplified with oCCS_90 and oCCS_91 using the same protocol as described in the library cloning method and subsequently Gibson assembled into BsmBI digested pCCS_103, which is a gRNA expression vector with a Hygromycin selection marker.

Stable polyclonal reporter lines were generated for each individual ORF by nucleofection of K562-Cas9-rtTA cells with 1 μg of ORF-EGFP-IRES-mCherry reporter construct and 500 ng of pCMV-hyPBase^11^. 2 days post-transfection, integrated cells were selected using 2 ug/mL puromycin for an additional 6 days.

In order to test the effect of individual E3 knockout on substrate abundance, cells expressing individual ORF-EGFP-IRES-mCherry reporters were transfected in triplicate with 750 ng of gRNA expression plasmid that contained a single unique gRNA sequence (either E3-specific gRNA or an NTC gRNA) along with 100 ng transposase expression construct. Cells were selected with 200 ug uL^−1^ Hygromycin B (Gibco) 2 days post-transfection and the EGFP:mCherry ratio was measured 8 days following transfection of the gRNA expression plasmid.

For the assessment of AlphaFold-Multimer nominated degrons, putative substrate degron regions were cloned into pCCS_196, which is a version of pCCS_82 where the Gateway cloning cassette was removed and multi-cloning sites were added at the 5’ and 3’ ends of the EGFP coding sequence. To clone degron sequences at the N-terminus of EGFP, pCCS_196 was digested with XhoI (NEB) and BamHI-HF (NEB) and the linearized backbone was purified by agarose gel purification (Monarch DNA Gel Extraction Kit, NEB). To clone degron sequences at the C-terminus of EGFP, pCCS_196 was digested with NheI-HF (NEB) and MluI-HF (NEB) and the linearized backbone was purified by agarose gel purification (Monarch DNA Gel Extraction Kit, NEB). DNA fragments encoding the putative degron regions were synthesized (gBlocks; IDT) with appropriate 5’ and 3’ homology to the digested backbone. These fragments were Gibson assembled into digested backbone (NEBuilder HiFi DNA Assembly, NEB) and transformed into *Escherichia coli* (NEB, C3040).

pCCS_196 degron reporters were then tested in cells using the same workflow described for the pCCS_82 reporter described at the top of this section.

##### Bioinformatic Analyses

###### ORF-barcode subassembly

In order to associate ORFs to DNA barcodes, sequencing libraries were prepared from barcoded COMET libraries (see “Subassembly of ORF-barcode pairs” section above). Reads from tagmented COMET libraries were aligned to ORF sequences using bowtie2 v2.2.5 to determine which ORF was tagged. A custom bowtie2 index of ORF sequences was generated and aligned against. A summary output format of the data was then generated that included the read name, the degenerate barcode sequence, the bowtie2-determined tagged ORF and the mapping quality of the read, which was then used as input for further QC to determine the list of BC-associated ORFs.

The initial list of barcodes and ORFs were filtered to exclude reads with a mapping quality score less than 31, barcodes that include any ‘N’ bases, and barcodes associated with common sequencing error modes (*e.g*., GGGGGGGGGGGGGGGGGGGGGGGGG). We then eliminated barcodes with fewer than 10 reads of supporting evidence, which guards against seemingly unique barcodes that actually include rarely occurring sequencing errors. Ambiguous barcodes were eliminated which mapped to more than one ORF, however, these were included if there was one ORF with more than 90% of the read evidence supporting one barcode. The remaining barcode-ORF pairs were then used to identify the ORF on a plasmid relying on the sequencing of just the barcode (Table S1).

###### Processing barcode-gRNA sequencing data

Amplicon sequencing reads of gDNA extracted from cells sorted by GFP:mCherry ratio contain two important pieces of information: the sequenced gRNA along with a barcode that was previously associated with a specific ORF. As a first step, we convert the raw read data into a summary output format that includes ORF (determined with the previously generated barcode-ORF lookup table), the target identity of the sequenced gRNA, along with the raw sequencing information of the barcode and guide. Summary output files from libraries generated from different conditions and sorted bins are then combined into one file that is then used for statistical analysis.

###### Screen analysis

The number of reads corresponding to each gRNA-barcode pair in each FACS bin and replicate were summed and used to calculate a Protein Stability Index (PSI) value for each pair (Table S2). PSI attempts to quantify the mean bin position for a gRNA-barcode pair by multiplying the bin number by the proportion of reads in that bin. The PSI ranges from 1 (low abundance) to 4 (high abundance).

After PSIs were computed per gRNA-barcode pair for each condition and replicate, we used a standard t-test to compare the distribution of PSI values between an ORF with a specific gRNA to the ORF with a non-targeting control gRNA. This test quantifies changes in PSI values per ORF explained by knockout of the targeted gene. To avoid additional variability of constructs with low representation, we filtered constructs with fewer than 50 reads per guide-barcode in the SCF experiment. For the short half-life TF experiment we filtered constructs with fewer than 100 reads per guide-barcode. Additionally, we removed ORF-target pairs with less than 15 barcodes to ensure sufficient observations for statistical testing. Finally, we also tested the effect of non-targeting control gRNAs on PSI distribution to estimate a null p-value distribution. We used Bonferroni and Benjamini-Hochberg approaches for multiple hypothesis correction and reported both values along with the raw p-value (Table S3). Significance was determined using the Benjamini-Hochberg corrected p-values.

###### Analysis of E3 ubiquitin ligase expression in K562

Files containing bulk poly-A RNA sequencing gene quantifications were downloaded from ENCODE^51^ (files ENCFF186TXT, ENCFF354ODN, ENCFF489VUK, ENCFF515MUX, ENCFF662LZE, ENCFF728TIT, ENCFF739YLB, ENCFF764ZIV, ENCFF930UOM, ENCFF934YBO). Gene quantifications for ubiquitin ligase components of interest were subset from the dataframe and ordered based on median FPKM (Figure S7).

###### *AlphaFold-Multimer* E3-substrate modeling

All computational protein models were run using a local installation of localcolabfold^52^ (https://github.com/YoshitakaMo/localcolabfold). E3-substrate pairs were modeled with *Alphafold-Multimer* (v3) using the --model-type alphafold2_multimer_v3 flag and monomers were modeled with *Alphafold2* using the --model-type alphafold2_ptm flag. The early stop tolerance was set to 0.5. Five models were generated with the number of recycles set to 30. Each model was then ranked by interface pTM (ipTM) to select the highest confidence model. Models were then AMBER relaxed for more accurate side chain predictions. Predicted models were visualized with ChimeraX.

###### Note on selection of E3-protein pairs from the BioPlex dataset

To obtain a large number of candidate E3-protein pairs with evidence of a physical interaction, we turned to the BioPlex dataset^40^, a proteome-scale map of experimentally derived protein-protein interactions generated via affinity-purification mass spectrometry. We specifically utilized the HEK293T dataset consisting of 118,162 PPIs. A list 568 E3s^53^ was used to isolate PPIs in which one of the interaction partners was an E3. To prevent artifacts from dimeric E3s, PPI pairs in which both partners were E3s were excluded, as well as any PPIs that involved an E3 and a core CRL subunit such as RBX1 or a cullin. Under these conditions we identified 7,581 BioPlex-supported E3-protein pairs with experimental evidence of a physical interaction, from which we randomly sampled 1,000 pairs to use for generating the positive control BioPlex *AlphaFold-Multimer* prediction set.

###### Extraction of prediction quality metrics from AlphaFold predictions

In order to globally access all predictions generated in the manuscript, we utilized a previously published python script^41^ (https://github.com/walterlab-HMS/AF2multimer-analysis; colabfold_analysis.py). The colabfold_analysis.py script takes *AlphaFold* output files and returns statistics such as the pLDDT per individual residue, the PAE value for each pair of residues (between and within individual chains), and the number of contacts. We ran the colabfold_analysis.py script on each prediction using the following parameters to define interchain contacts as follows: **1)** a maximum distance of 5 Angstroms between any two atoms in two residues (--distance 5), **2)** a minimum PAE value of 10 between each residue in the contact (--pae 10 and --pae-mod min), and **3)** a minimum pLDDT value >=50 for both residues (--plddt 50). The resulting output files containing residues meeting the above criteria were then used to assess prediction quality as described below.

Mean PAE values for all contacts in each model were calculated and subsequently normalized between 0 and 1 using all prediction classes (COMET-nominated, screen-negative and true random). More specifically, the inverse of the normalized PAE values was taken so that the best values of the resulting metric were close to 1. Information about contact consistency across models was incorporated by quantifying the average number of models each contact was observed in (minimum = 1, maximum = 5) for each complex, and these values were also normalized between 0 and 1 using all prediction classes.

A weighted sum incorporating the normalized PAE and contact consistency values was then used to compute a summary score as follows:

$$S=\left(PAE_{\text{avg}}\cdot \frac{1}{2}\right)+\left({\left(\frac{\;\text{models}}{\;\text{contact}}\right)}_{\text{avg}}\cdot \frac{1}{2}\right)$$

### QUANTIFICATION AND STATISTICAL ANALYSIS

Statistical tests for each experiment are described in the text, figure legends or STAR Methods. In Figures 2D, 2E, 3E, 4F, S3C, S3D, S3E, S4A, S4B, Pearson’s correlation coefficients were calculated using the cor() function in R. In Figures S3C, S3D, S3E the fitted line was produced using function geom_smooth() in the R package ggplot using parameter ‘method = “lm”’ and all other parameters being default. Error bars in Figures 3D, 4D, and all panels of Figure S7 correspond to standard error. For all COMET screens (Figures 3F,H, 4E,F), PSIs were compared between NTC and target genes using the t.test() function in R, and the *p* values were adjusted via the Benjamini–Hochberg procedure using the p.adjust() function with the method set to Benjamini-Hochberg (method=‘BH’). In Figure 3G, a one-sided hypergeometric test was computed using the p.hyper() function in R with the lower.tail = FALSE parameter specified. In Figure 5E, Bonferroni corrected *p* values were calculated using a two-sided pairwise Wilcoxon test via the pairwise.wilcox.test() function in R with the p.adjust.method parameter set to Bonferroni (p.adjust.method = “bonferroni”). In Figure 5G, a two-sided Fisher’s exact test was used to identify differences in the proportions of complexes that pass the threshold using fisher.test() function in R. Resulting *p* values were adjusted in R using the p.adjust() function with the method set to Benjamini-Hochberg (method=‘BH’). In Figure S7, the mean of the NTC and gRNA GFP:mCherry ratios were compared using a one-sided t-test using the t.test() function in R with the alternative hypothesis set to greater (alternative = “greater”).

## Supplementary Material

1 **Document S1**. Figures S1–S7

2 **Table S1:** ORF-barcode associations for each library, related to Figures 2, 3, and 4.

3 **Table S2:** Computed PSIs for each ORF-target pair in each experiment, related to Figures 2, 3, and 4.

4 **Table S3:** Full screen results for each experiment, related to Figures 3 and 4.

5 **Table S4:** Connection classes for E3-substrate relationships, related to Figures 3 and 4.

6 **Table S5**: Extracted contact data for all *AlphaFold-Multimer* models, related to Figure 6.

7 **Table S6**: Oligonucleotides used in this manuscript, related to STAR Methods.

## Figures and Tables

**Key resources table:** 

REAGENT or RESOURCE	SOURCE	IDENTIFIER
Bacterial and virus strains		
NEB 10-beta Electrocompetent *E. coli*	New England Biolabs	C3020K
NEB Stable Competent E. coli	New England Biolabs	C3040H
Chemicals, peptides, and recombinant proteins		
RPMI 1640 Medium	Gibco	11875119
DMEM, High Glucose	Gibco	11965118
FBS	Hyclone	SH30396.03
Penicillin-Streptomycin (10,000 U/mL)	Gibco	15140122
DNeasy Blood & Tissue Kit	QIAGEN	69504
AMPure XP Reagent	Beckman Coulter Life Sciences	A63882
KAPA HiFi HotStart ReadyMix	Roche	KK2602
KAPA2G Robust HotStart ReadyMix PCR Kit	Roche	KK5702
NEBNext High-Fidelity 2X PCR Master Mix	New England Biolabs	M0541S
NEBuilder HiFi DNA Assembly Cloning Kit	New England Biolabs	E5520S
Gateway LR Clonase II Enzyme mix	Invitrogen	11791020
Blasticidin S HCl (10 mg/mL)	Gibco	A1113903
Geneticin	Gibco	10131035
Puromycin Dihydrochloride	Gibco	A1113803
OC-100x2 processing assembly	MaxCyte	SOC-1x2
SF Cell Line 4D-Nucleofector X Kit	Lonza Bioscience	V4XC-2012
Lipofectamine 3000 Transfection	Invitrogen	L3000001
Reagent		
Tn5 transposase	Diagenode	C01070010–20
DNA Polymerase I, Large (Klenow) Fragment	New England Biolabs	M0210S
I-SceI	New England Biolabs	R0694S
I-CeuI	New England Biolabs	R0699S
rCutSmart Buffer	New England Biolabs	B6004S
Recombinant Albumin, Molecular Biology Grade	New England Biolabs	B9200S
dNTP mix	New England Biolabs	N0447L
BsmBI-v2	New England Biolabs	R0739S
XhoI	New England Biolabs	R0146S
BamHI-HF	New England Biolabs	R3136S
NheI-HF	New England Biolabs	R3131S
MluI-HF	New England Biolabs	R3198S
Critical commercial assays		
NextSeq 1000/2000 P2 Reagents (100 Cycles)	Illumina	20046811
NextSeq 1000/2000 P2 Reagents (200 cycles)	Illumina	20046812
Deposited data		
Sequencing data generated in this study	This manuscript	GEO: GSE234621
K562 bulk RNA-seq data	ENCODE Project Consortium	ENCFF186TXT; ENCFF354ODN, ENCFF489VUK; ENCFF515MUX; ENCFF662LZE; ENCFF728TIT; ENCFF739YLB; ENCFF764ZIV; ENCFF930UOM; ENCFF934YBO
Experimental models: Cell lines		
K562	ATCC	CCL-243
HEK293	ATCC	CRL-1573
K652-Cas9-rtTA monoclonal line	This manuscript	N/A
HEK293-Cas9-rtTA monoclonal line	This manuscript	N/A
Oligonucleotides		
List of oligonucleotides	This manuscript; Table S5	NA
Recombinant DNA		
pCMV-HyPBase	Yusa et al.	NA
pB-rtTA	Addgene	#126034
pCCS_11; TRE Promoter donor plasmid; pUC19-I-SceI-tight-TRE-I-CeuI	This manuscript	NA
pCCS_30; Parental COMET backbone; PB-Puro-U6-Stuffer-Scaffold-[I-SceI / PI-SceI / I-CeuI]-attR1-CmR-ccdB-attR2-EGFP-IRES-mCherry-SV40	This manuscript	NA
pCCS_82; Validation Reporter; PB-Puro-TRE-attR1-CmR-ccdB-attR2-EGFP-IRES-mCherry-SV40	This manuscript	NA
pCCS_103; gRNA expression plasmid; pB-U6-BsmBI-Scaffold-Hygro	This manuscript	NA
pCCS_196; Degron Validation Reporter; PB-Puro-TRE-MCS-EGFP-MCS-IRES-mCherry-SV40	This manuscript	NA
Software and algorithms		
Bcl2fastq (v2.20)	Illumina	https://support.illumina.com/sequencing/sequencing_software/bcl2fastqconversion-software.html
localcolabfold (1.5.5)	Mirdita et al	https://github.com/YoshitakaMo/localcolabfold
AlphaFold (alphafold2_ptm)	Jumper et al	https://github.com/google-deepmind/alphafold
AlphaFold-multimer (v3)	Evans et al	https://github.com/google-deepmind/alphafold
AF2multimer-analysis	Lim et al	https://github.com/walterlab-HMS/AF2multimer-analysis

## Highlights

- Developed a combinatorial assay to test E3-substrate interactions at scale
- Identified known and unknown E3-substrate relationships across three screens
- Assessment of *in silico* models points to scalable computational substrate discovery
- Computed models of E3-substrate interactions reveal known and putative degron motifs

## References

[1] King RW, Deshaies RJ, Peters JM, and Kirschner MW (1996). How proteolysis drives the cell cycle. Science 274, 1652–1659. 8939846 10.1126/science.274.5293.1652

[2] Tan X, Calderon-Villalobos LIA, Sharon M, Zheng C, Robinson CV, Estelle M, and Zheng N (2007). Mechanism of auxin perception by the TIR1 ubiquitin ligase. Nature 446, 640–645. 17410169 10.1038/nature05731

[3] Berndsen CE, and Wolberger C (2014). New insights into ubiquitin E3 ligase mechanism. Nat. Struct. Mol. Biol. 21, 301–307. 24699078 10.1038/nsmb.2780

[4] Deshaies RJ, and Joazeiro CAP (2009). RING domain E3 ubiquitin ligases. Annu. Rev. Biochem. 78, 399–434. 19489725 10.1146/annurev.biochem.78.101807.093809

[5] Iconomou M, and Saunders DN (2016). Systematic approaches to identify E3 ligase substrates. Biochem. J 473, 4083–4101. 27834739 10.1042/BCJ20160719 PMC5103871

[6] Pierce NW, Kleiger G, Shan S-O, and Deshaies RJ (2009). Detection of sequential polyubiquitylation on a millisecond timescale. Nature 462, 615–619. 19956254 10.1038/nature08595 PMC2791906

[7] Yen H-CS, and Elledge SJ (2008). Identification of SCF ubiquitin ligase substrates by global protein stability profiling. Science 322, 923–929. 18988848 10.1126/science.1160462

[8] Petroski MD, and Deshaies RJ (2005). Function and regulation of cullin–RING ubiquitin ligases. Nat. Rev. Mol. Cell Biol. 6, 9–20. 15688063 10.1038/nrm1547

[9] Yen H-CS, Xu Q, Chou DM, Zhao Z, and Elledge SJ (2008). Global protein stability profiling in mammalian cells. Science 322, 918–923. 18988847 10.1126/science.1160489

[10] Jin J, Cardozo T, Lovering RC, Elledge SJ, Pagano M, and Harper JW (2004). Systematic analysis and nomenclature of mammalian F-box proteins. Genes Dev. 18, 2573–2580. 15520277 10.1101/gad.1255304 PMC525538

[11] Yusa K, Zhou L, Li MA, Bradley A, and Craig NL (2011). A hyperactive piggyBac transposase for mammalian applications. Proc. Natl. Acad. Sci. U. S. A. 108, 1531–1536. 21205896 10.1073/pnas.1008322108 PMC3029773

[12] Emanuele MJ, Elia AEH, Xu Q, Thoma CR, Izhar L, Leng Y, Guo A, Chen Y-N, Rush J, Hsu PW-C, (2011). Global identification of modular cullin-RING ligase substrates. Cell 147, 459–474. 21963094 10.1016/j.cell.2011.09.019 PMC3226719

[13] Dankert JF, Rona G, Clijsters L, Geter P, Skaar JR, Bermudez-Hernandez K, Sassani E, Fenyö D, Ueberheide B, Schneider R, (2016). Cyclin F-Mediated Degradation of SLBP Limits H2A.X Accumulation and Apoptosis upon Genotoxic Stress in G2. Mol. Cell 64, 507–519. 27773672 10.1016/j.molcel.2016.09.010 PMC5097008

[14] Cui D, Xiong X, Shu J, Dai X, Sun Y, and Zhao Y (2020). FBXW7 Confers Radiation Survival by Targeting p53 for Degradation. Cell Rep. 30, 497–509.e4. 31940492 10.1016/j.celrep.2019.12.032

[15] Kimura T, Gotoh M, Nakamura Y, and Arakawa H (2003). hCDC4b, a regulator of cyclin E, as a direct transcriptional target of p53. Cancer Sci. 94, 431–436. 12824889 10.1111/j.1349-7006.2003.tb01460.x PMC11160198

[16] Williams AB, and Schumacher B (2016). p53 in the DNA-Damage-Repair Process. Cold Spring Harb. Perspect. Med. 6. 10.1101/cshperspect.a026070. PMC4852800 27048304

[17] Vicente C, Conchillo A, García-Sánchez MA, and Odero MD (2012). The role of the GATA2 transcription factor in normal and malignant hematopoiesis. Crit. Rev. Oncol. Hematol. 82, 1–17. 21605981 10.1016/j.critrevonc.2011.04.007

[18] Nakajima T, Kitagawa K, Ohhata T, Sakai S, Uchida C, Shibata K, Minegishi N, Yumimoto K, Nakayama KI, Masumoto K, (2015). Regulation of GATA-binding protein 2 levels via ubiquitin-dependent degradation by Fbw7: involvement of cyclin B-cyclin-dependent kinase 1-mediated phosphorylation of THR176 in GATA-binding protein 2. J. Biol. Chem. 290, 10368–10381. 25670854 10.1074/jbc.M114.613018 PMC4400347

[19] Lurie LJ, Boyer ME, Grass JA, and Bresnick EH (2008). Differential GATA Factor Stabilities: Implications for Chromatin Occupancy by Structurally Similar Transcription Factors. Biochemistry 47, 859–869. 18154321 10.1021/bi701692p

[20] van der Harst P, Zhang W, Mateo Leach I, Rendon A, Verweij N, Sehmi J, Paul DS, Elling U, Allayee H, Li X, (2012). Seventy-five genetic loci influencing the human red blood cell. Nature 492, 369–375. 23222517 10.1038/nature11677 PMC3623669

[21] Randle SJ, Nelson DE, Patel SP, and Laman H (2015). Defective erythropoiesis in a mouse model of reduced Fbxo7 expression due to decreased p27 expression. J. Pathol. 237, 263–272. 26095538 10.1002/path.4571 PMC4583784

[22] Mark KG, and Rape M (2021). Ubiquitin-dependent regulation of transcription in development and disease. EMBO Rep. 22, e51078. 33779035 10.15252/embr.202051078 PMC8025022

[23] Lipford JR, and Deshaies RJ (2003). Diverse roles for ubiquitin-dependent proteolysis in transcriptional activation. Nat. Cell Biol. 5, 845–850. 14523392 10.1038/ncb1003-845

[24] Geng F, Wenzel S, and Tansey WP (2012). Ubiquitin and proteasomes in transcription. Annu. Rev. Biochem. 81, 177–201. 22404630 10.1146/annurev-biochem-052110-120012 PMC3637986

[25] Kronke J, Udeshi ND, Narla A, Grauman P, Hurst SN, McConkey M, Svinkina T, Heckl D, Comer E, Li X, (2014). Lenalidomide Causes Selective Degradation of IKZF1 and IKZF3 in Multiple Myeloma Cells. Preprint, https://doi.org/10.1126/science.1244851 10.1126/science.1244851. PMC4077049 24292625

[26] Lu G, Middleton RE, Sun H, Naniong M, Ott CJ, Mitsiades CS, Wong K-K, Bradner JE, and Kaelin WG Jr (2014). The myeloma drug lenalidomide promotes the cereblon-dependent destruction of Ikaros proteins. Science 343, 305–309. 24292623 10.1126/science.1244917 PMC4070318

[27] Vassilev LT, Vu BT, Graves B, Carvajal D, Podlaski F, Filipovic Z, Kong N, Kammlott U, Lukacs C, Klein C, (2004). In vivo activation of the p53 pathway by small-molecule antagonists of MDM2. Science 303, 844–848. 14704432 10.1126/science.1092472

[28] Li J, Cai Z, Vaites LP, Shen N, Mitchell DC, Huttlin EL, Paulo JA, Harry BL, and Gygi SP (2021). Proteome-wide mapping of short-lived proteins in human cells. Mol. Cell 81, 4722–4735.e5. 34626566 10.1016/j.molcel.2021.09.015 PMC8892350

[29] Peters J-M (2006). The anaphase promoting complex/cyclosome: a machine designed to destroy. Nat. Rev. Mol. Cell Biol. 7, 644–656. 16896351 10.1038/nrm1988

[30] Clute P, and Pines J (1999). Temporal and spatial control of cyclin B1 destruction in metaphase. Nat. Cell Biol. 1, 82–87. 10559878 10.1038/10049

[31] Hagting A, Den Elzen N, Vodermaier HC, Waizenegger IC, Peters J-M, and Pines J (2002). Human securin proteolysis is controlled by the spindle checkpoint and reveals when the APC/C switches from activation by Cdc20 to Cdh1. J. Cell Biol. 157, 1125–1137. 12070128 10.1083/jcb.200111001 PMC2173548

[32] Glotzer M, Murray AW, and Kirschner MW (1991). Cyclin is degraded by the ubiquitin pathway. Nature 349, 132–138. 1846030 10.1038/349132a0

[33] Pfleger CM, and Kirschner MW (2000). The KEN box: an APC recognition signal distinct from the D box targeted by Cdh1. Genes Dev. 14, 655–665. 10733526 PMC316466

[34] Guharoy M, Bhowmick P, Sallam M, and Tompa P (2016). Tripartite degrons confer diversity and specificity on regulated protein degradation in the ubiquitin-proteasome system. Nat. Commun. 7, 1–13. 10.1038/ncomms10239 PMC4729826 26732515

[35] Südbeck P, and Scherer G (1997). Two Independent Nuclear Localization Signals Are Present in the DNA-binding High-mobility Group Domains of SRY and SOX9*. J. Biol. Chem. 272, 27848–27852. 9346931 10.1074/jbc.272.44.27848

[36] Gasca S, Canizares J, De Santa Barbara P, Mejean C, Poulat F, Berta P, and Boizet-Bonhoure B (2002). A nuclear export signal within the high mobility group domain regulates the nucleocytoplasmic translocation of SOX9 during sexual determination. Proc. Natl. Acad. Sci. U. S. A. 99, 11199–11204. 12169669 10.1073/pnas.172383099 PMC123233

[37] Cenik BK, and Shilatifard A (2020). COMPASS and SWI/SNF complexes in development and disease. Nat. Rev. Genet. 22, 38–58. 32958894 10.1038/s41576-020-0278-0

[38] Evans R, O’Neill M, Pritzel A, Antropova N, Senior A, Green T, Žídek A, Bates R, Blackwell S, Yim J, (2022). Protein complex prediction with AlphaFold-Multimer. bioRxiv, 2021.10.04.463034. 10.1101/2021.10.04.463034.

[39] Jumper J, Evans R, Pritzel A, Green T, Figurnov M, Ronneberger O, Tunyasuvunakool K, Bates R, Žídek A, Potapenko A, (2021). Highly accurate protein structure prediction with AlphaFold. Nature 596, 583–589. 34265844 10.1038/s41586-021-03819-2 PMC8371605

[40] Huttlin EL, Bruckner RJ, Navarrete-Perea J, Cannon JR, Baltier K, Gebreab F, Gygi MP, Thornock A, Zarraga G, Tam S, (2021). Dual proteome-scale networks reveal cell-specific remodeling of the human interactome. Cell 184, 3022–3040.e28. 33961781 10.1016/j.cell.2021.04.011 PMC8165030

[41] Lim Y, Tamayo-Orrego L, Schmid E, Tarnauskaite Z, Kochenova OV, Gruar R, Muramatsu S, Lynch L, Schlie AV, Carroll PL, (2023). In silico protein interaction screening uncovers DONSON’s role in replication initiation. Science 381, eadi3448. 37590370 10.1126/science.adi3448 PMC10801813

[42] Simoneschi D, Rona G, Zhou N, Jeong Y-T, Jiang S, Milletti G, Arbini AA, O’Sullivan A, Wang AA, Nithikasem S, (2021). CRL4AMBRA1 is a master regulator of D-type cyclins. Nature 592, 789–793. 33854235 10.1038/s41586-021-03445-y PMC8875297

[43] Puklowski A, Homsi Y, Keller D, May M, Chauhan S, Kossatz U, Grünwald V, Kubicka S, Pich A, Manns MP, (2011). The SCF–FBXW5 E3-ubiquitin ligase is regulated by PLK4 and targets HsSAS-6 to control centrosome duplication. Nat. Cell Biol. 13, 1004–1009. 21725316 10.1038/ncb2282

[44] Werner A, Disanza A, Reifenberger N, Habeck G, Becker J, Calabrese M, Urlaub H, Lorenz H, Schulman B, Scita G, (2013). SCFFbxw5 mediates transient degradation of actin remodeller Eps8 to allow proper mitotic progression. Nat. Cell Biol. 15, 179–188. 23314863 10.1038/ncb2661 PMC3749308

[45] Zhang C, Li X, Adelmant G, Dobbins J, Geisen C, Oser MG, Wucherpfenning KW, Marto JA, and Kaelin WG (2015). Peptidic degron in EID1 is recognized by an SCF E3 ligase complex containing the orphan F-box protein FBXO21. Proceedings of the National Academy of Sciences 112, 15372–15377. 10.1073/pnas.1522006112 PMC4687553 26631746

[46] Morreale FE, and Walden H (2016). Types of Ubiquitin Ligases. Cell 165, 248–248.e1. 27015313 10.1016/j.cell.2016.03.003

[47] Tokheim C, Wang X, Timms RT, Zhang B, Mena EL, Wang B, Chen C, Ge J, Chu J, Zhang W, (2021). Systematic characterization of mutations altering protein degradation in human cancers. Mol. Cell 81, 1292–1308.e11. 33567269 10.1016/j.molcel.2021.01.020 PMC9245451

[48] Timms RT, Mena EL, Leng Y, Li MZ, Tchasovnikarova IA, Koren I, and Elledge SJ (2023). Defining E3 ligase–substrate relationships through multiplex CRISPR screening. Nat. Cell Biol. 25, 1535–1545. 37735597 10.1038/s41556-023-01229-2 PMC10567573

[49] Lazar NH, Celik S, Chen L, Fay M, Irish JC, Jensen J, Tillinghast CA, Urbanik J, Bone WP, Roberts GHL, (2023). High-resolution genome-wide mapping of chromosome-arm-scale truncations induced by CRISPR-Cas9 editing. bioRxiv, 2023.04.15.537038. 10.1101/2023.04.15.537038. PMC11250378 38811841

[50] Doench JG, Fusi N, Sullender M, Hegde M, Vaimberg EW, Donovan KF, Smith I, Tothova Z, Wilen C, Orchard R, (2016). Optimized sgRNA design to maximize activity and minimize off-target effects of CRISPR-Cas9. Nat. Biotechnol. 34, 184–191. 26780180 10.1038/nbt.3437 PMC4744125

[51] ENCODE Project Consortium (2012). An integrated encyclopedia of DNA elements in the human genome. Nature 489, 57–74. 22955616 10.1038/nature11247 PMC3439153

[52] Mirdita M, Schütze K, Moriwaki Y, Heo L, Ovchinnikov S, and Steinegger M (2022). ColabFold: making protein folding accessible to all. Nat. Methods 19, 679–682. 35637307 10.1038/s41592-022-01488-1 PMC9184281

[53] Słabicki M, Kozicka Z, Petzold G, Li Y-D, Manojkumar M, Bunker RD, Donovan KA, Sievers QL, Koeppel J, Suchyta D, (2020). The CDK inhibitor CR8 acts as a molecular glue degrader that depletes cyclin K. Nature 585, 293–297. 32494016 10.1038/s41586-020-2374-x PMC7486275

