# Supplementary material for: Combinatorial mapping of E3 ubiquitin ligases to their target substrates
Source: Mol Cell. Author manuscript; Available in PMC 2025 Feb 22. (PMC11845296; doi:10.1016/j.molcel.2025.01.016)
Supplement: 1 — Document S1. Figures S1–S7 [file NIHMS2050009-supplement-1.pdf]

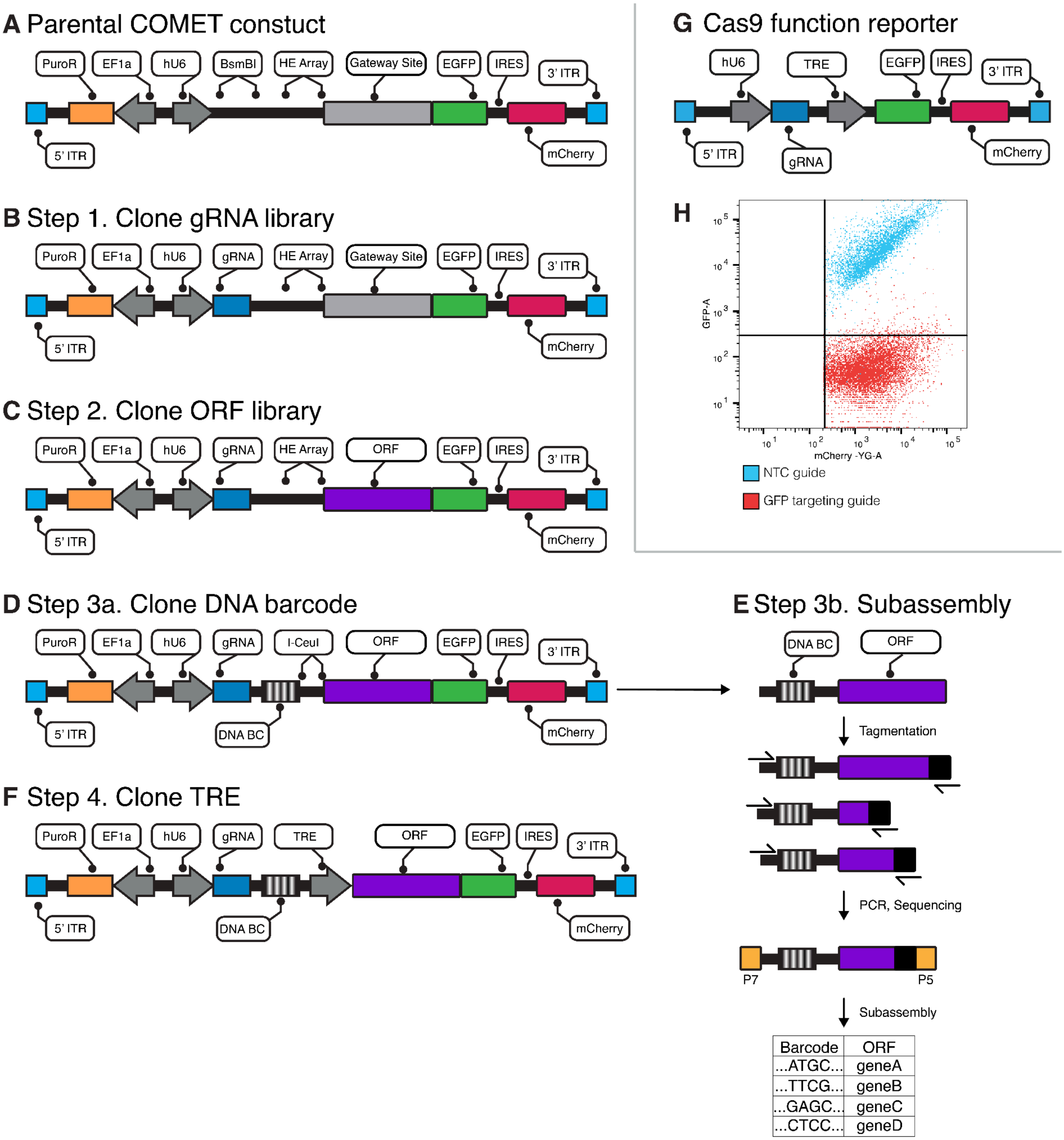

Barcode	ORF
...ATGC...	geneA
...TTCG...	geneB
...GAGC...	geneC
...CTCC...	geneD

Figure S1. Schematic of COMET library cloning workflow and Cas9 function reporter, related to STAR Methods.

A-F) The parental COMET construct (**A**) is digested with BsmB**I** and a gRNA library is cloned into the backbone (**B**). ORF libraries are then recombined into the gRNA containing plasmid library (**C**). The gRNA and ORF containing library is then digested with two homing endonucleases (targeting the HE Array shown in Step 2 schematic), and a 25mer DNA oligo is cloned into the backbone in order to barcode the library (**D**). These barcodes are associated to ORFs via subassembly (**E**). Finally, the barcoded gRNA and ORF library is digested at dual **I**-Ceu**I** sites and a tetracycline response element (TRE) promoter is cloned into the backbone (**F**). **G)** Schematic of reporter used to test for cell line reporter induction as well as Cas9 activity. Two versions of this reporter were cloned, one with a GFP-specific gRNA and one with an NTC gRNA. **H)** Flow cytometry plot of K562-rtTA-Cas9 clone 3 cells transfected with the GFP-specific gRNA containing reporter (red) as well as the NTC gRNA containing reporter (blue).

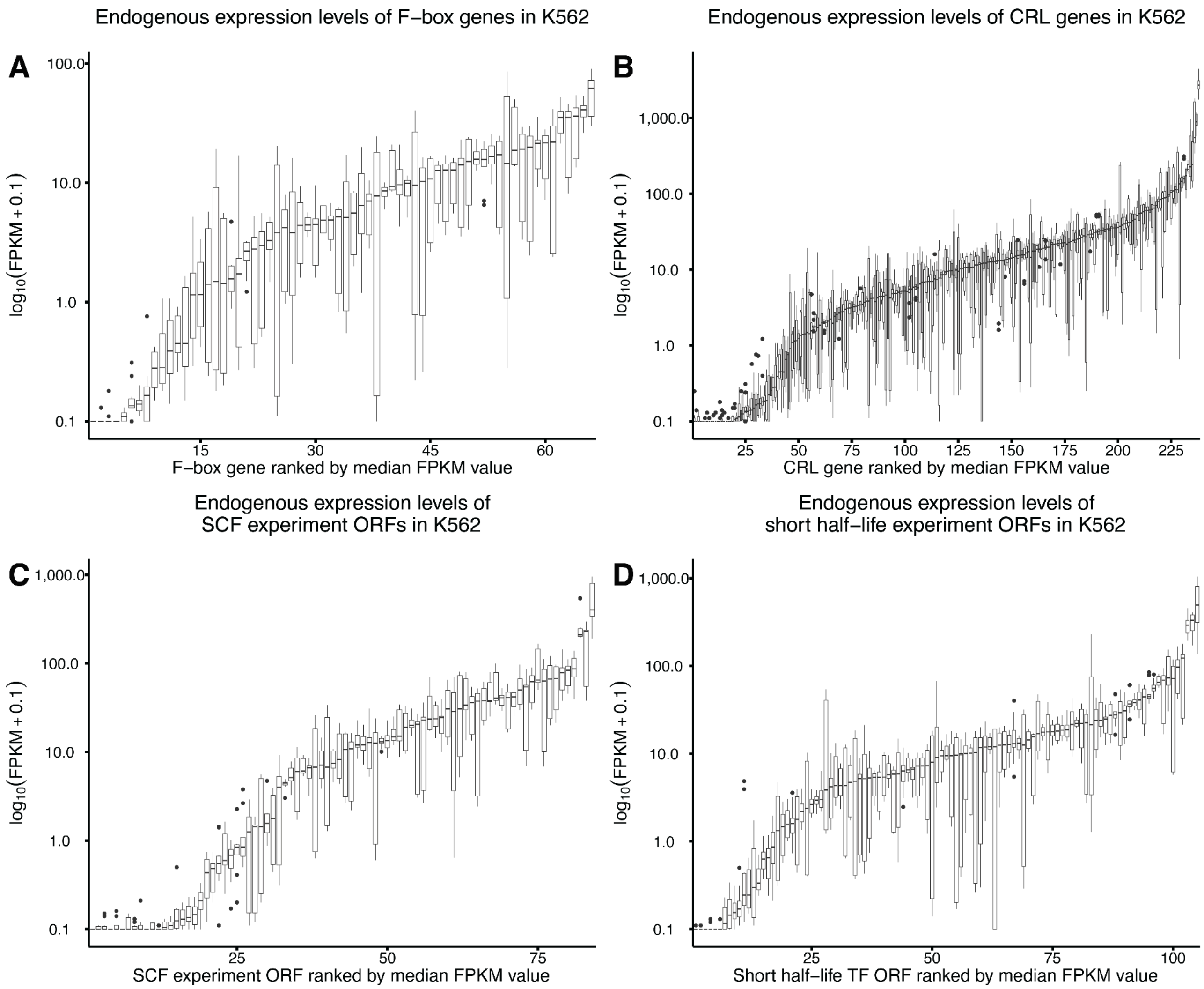

Figure S2: E3 ubiquitin ligase and substrate gene expression levels in K562 cells, related to Figures 3 and 4.

A) Boxplots showing endogenous gene expression levels in K562 cells of 68 F-box genes. Genes are ranked by median fragments per kilobase of transcript per million fragments mapped (FPKM). **B)** Boxplots showing endogenous gene expression levels in K562 cells of 241 CRL genes, ranked by median FPKM. **C)** Boxplots showing endogenous gene expression levels of the 92 putative substrate-endocoding ORFs from the SCF experiment in K562 cells. **D)** Boxplots showing endogenous gene expression levels in K562 cells of the 108 putative substrate-encoding ORFs tested in the short half-life TF experiment.

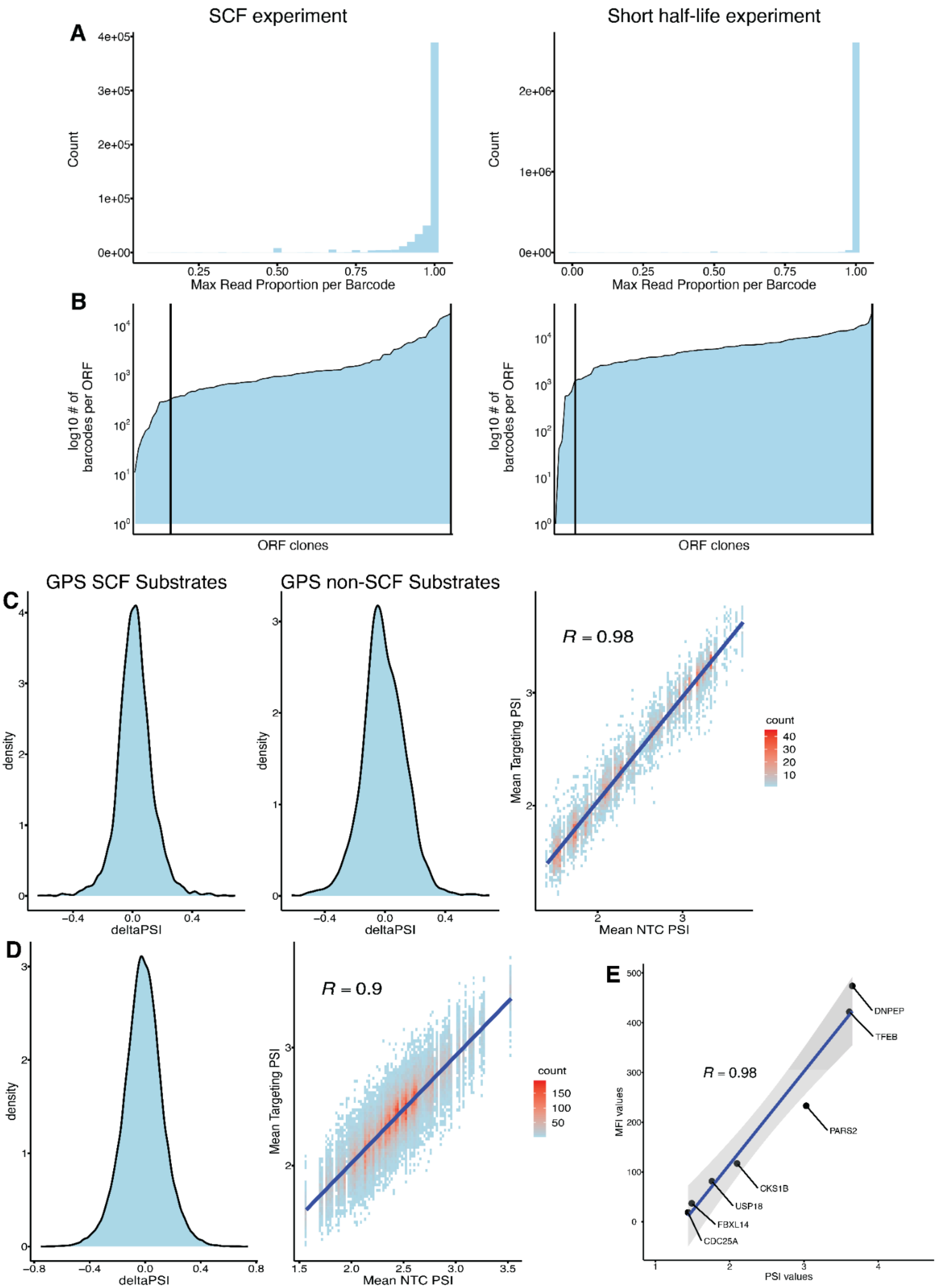

A
SCF experiment
Short half-life experiment
Count
Max Read Proportion per Barcode
4e+05
3e+05
2e+05
1e+05
0e+00
2e+06
1e+06
0.00
0.25
0.50
0.75
1.00
B
log10 # of barcodes per ORF
ORF clones
C
GPS SCF Substrates
GPS non-SCF Substrates
density
deltaPSI
R = 0.98
Mean Targeting PSI
Mean NTC PSI
count
D
R = 0.9
E
MFI values
PSI values
DNPEP
TFEB
PARS2
CKS1B
USP18
FBXL14
CDC25A

Figure S3: Visualization of barcode specificity, PSI distributions, and PSI/MFI correlation, related to Figures 3 and 4.

A) Histograms of the max read proportion of individual barcodes for the SCF (left) and short half-life experiments (right). For an individual barcode, the proportion of reads associated to any ORF was computed, and the vast majority (92% SCF, 98%) of barcodes associate to a single ORF >90% of the time. **B)** Line plots of the number of barcodes per ORF for the SCF (left) and short half-life experiments (right). Vertical black lines indicate ORFs with > 300 barcodes (left, SCF experiment) or > 1300 barcodes (right, short half-life experiment). **C)** Density plot of SCF experiment PSIs for GPS-annotated SCF substrate proteins (left) as well as proteins not identified as SCF substrates (middle). Scatter plot colored by point density (right) showing correlation between PSIs calculated for NTC gRNAs (x-axis) or targeting gRNAs (y-axis). **D)** Same as panel **C,** but for the short half-life TF experiment. **E)** Scatterplot comparison of flow cytometry derived Mean Fluorescence Intensities (MFI; y-axis) and computationally calculated Protein Stability Indexes (PSI; x-axis). R denotes Pearson's correlation coefficient.

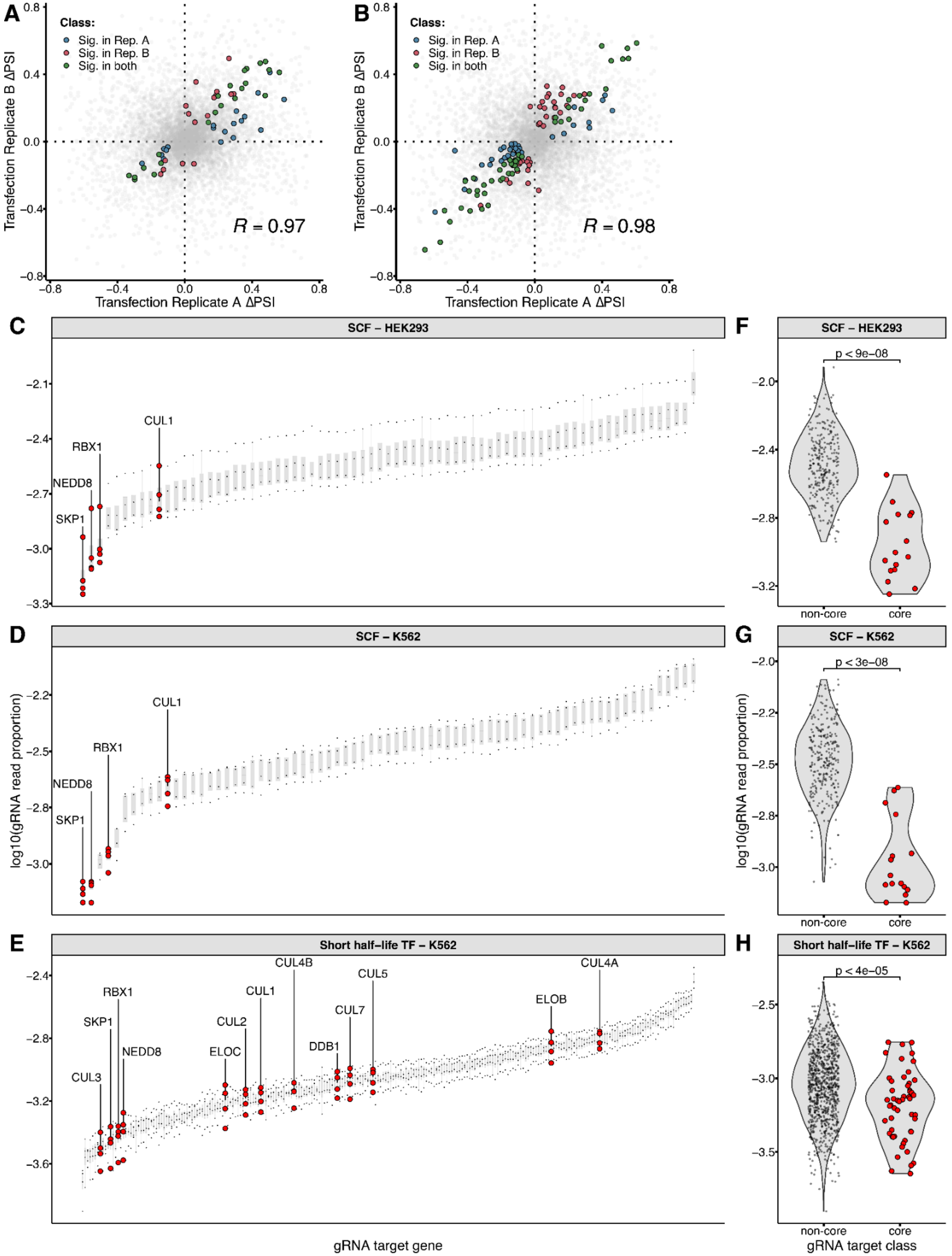
A
B
Class:
Sig. in Rep. A
Sig. in Rep. B
Sig. in both
Transfection Replicate B ΔPSI
Transfection Replicate A ΔPSI
R = 0.97
R = 0.98
0.8
0.4
0.0
−0.4
−0.8
C
SCF – HEK293
CUL1
RBX1
NEDD8
SKP1
−2.1
−2.4
−2.7
−3.0
−3.3
D
SCF – K562
CUL1
RBX1
NEDD8
SKP1
−2.2
−2.5
−2.8
−3.0
log10(gRNA read proportion)
E
Short half−life TF – K562
CUL3
SKP1
RBX1
NEDD8
ELOC
CUL2
CUL1
CUL4B
DDB1
CUL7
CUL5
ELOB
CUL4A
−2.4
−2.8
−3.2
−3.6
gRNA target gene
F
SCF – HEK293
p < 9e−08
−2.0
−2.4
−2.8
−3.2
G
SCF – K562
p < 3e−08
−2.0
−2.2
−2.5
−2.8
−3.0
H
Short half−life TF – K562
p < 4e−05
−2.5
−3.0
−3.5
non-core
core
gRNA target class

Figure S4: Assessment of ∆PSI correlation in two cell lines and core CRL subunit essentiality, related to Figures 3 and 4.

A) Reproducibility of ∆PSI estimates between collapsed replicates in HEK293 cells from the pilot SCF COMET experiment. Reads from four independent transfection replicates were collapsed into two replicates, and ∆PSIs and significance were recalculated. Points are colored based on significance in Replicate A (blue), replicate B (red), or both (green). Pearson correlation was calculated based on the subset of interactions that were significant in either or both cell lines. **B)** Same as panel **A** but for K562 cell line. **C-E)** Plots of gRNA counts isolated from genomic DNA 12 days after library transfection for the HEK293 SCF (**C**), K562 SCF (**D**), and the K562 short half-life TF (**E**) screens. Core SCF components such as cullin scaffolds and substrate adaptors are labeled and read proportions from four independent transfections replicates are indicated with red points. **F-H)** Read proportions for gRNAs targeting core CRL components were significantly different than read proportions for gRNAs targeting non-core CRL subunits. Significance was calculated using a two-sided t-test.

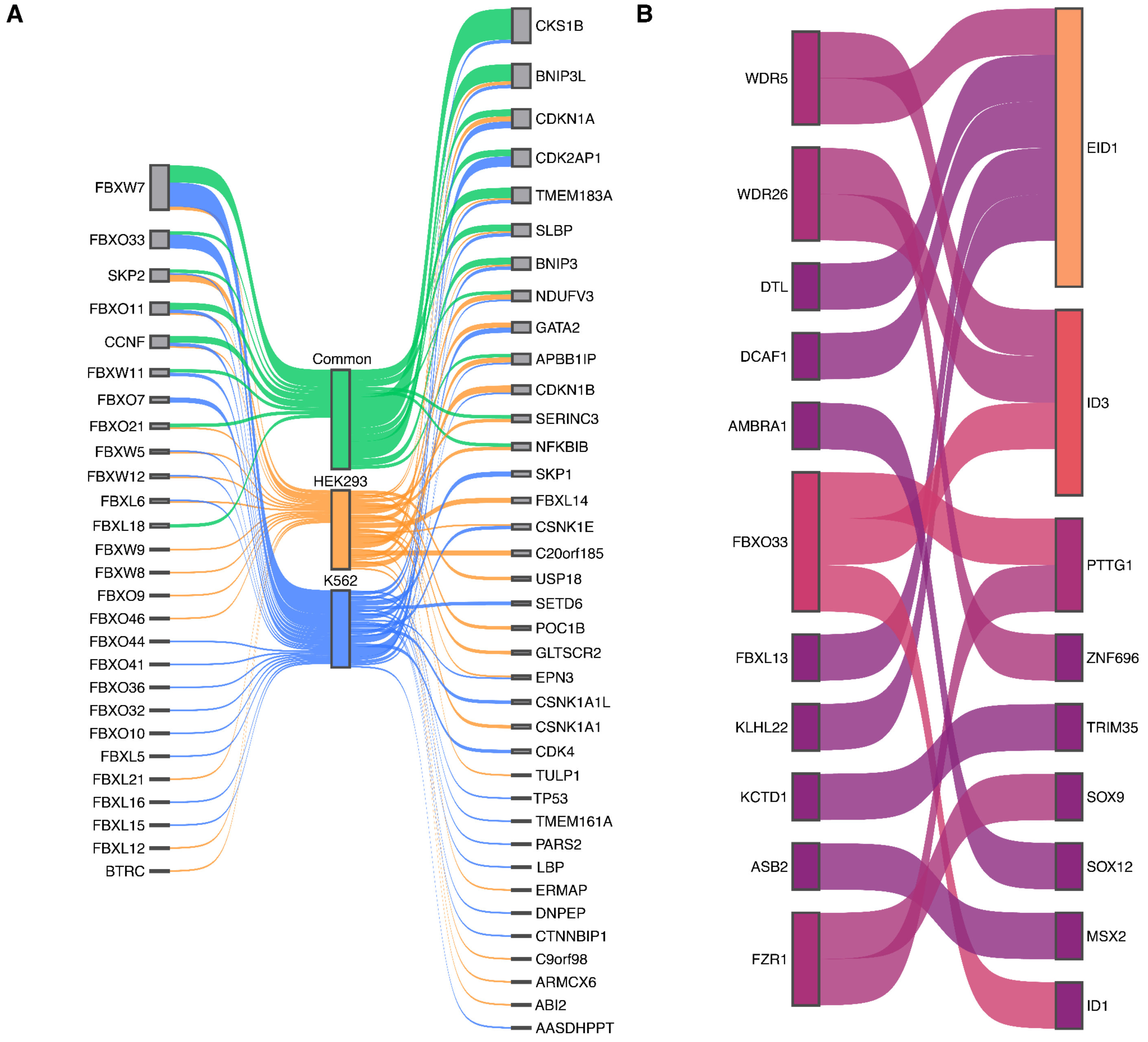

Figure S5: COMET maps E3 ligases to their target substrates, related to Figures 3 and 4.

A) Visual summary of results from the SCF experiment. E3s listed on the left are connected to all COMET-identified significant target substrates (p < 0.05, ΔPSI > 0). Flow colors correspond to the cell line in which the association was identified (orange = HEK293, blue = K562, and common = green). **B)** Same as panel **A**, except visually summarizing results from the TF experiment. Flow colors originating from the left correspond to the number of substrates linked to each E3.

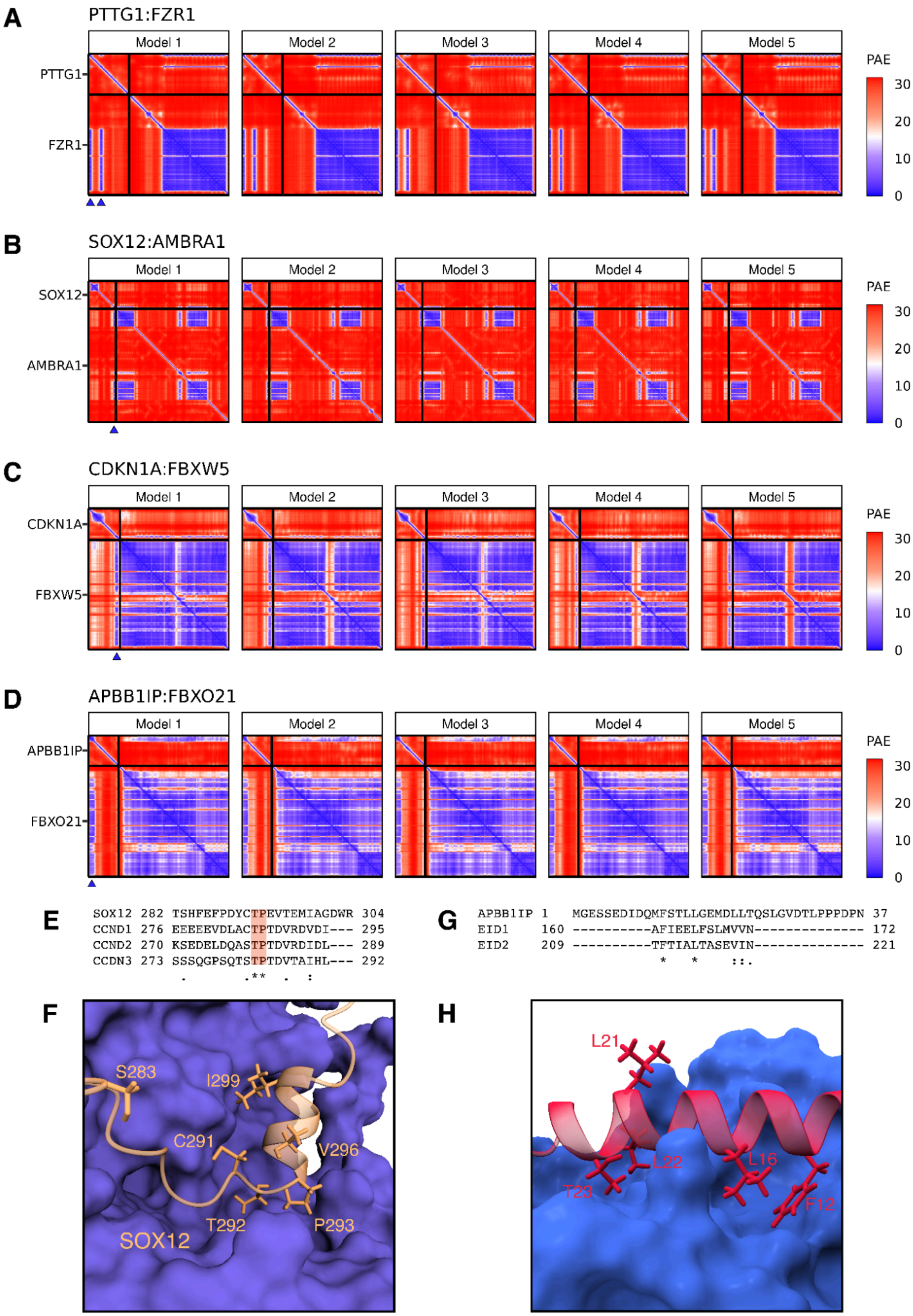

A
PTTG1:FZR1
Model 1
Model 2
Model 3
Model 4
Model 5
PTTG1
FZR1
PAE
30
20
10
0
B
SOX12:AMBRA1
SOX12
AMBRA1
C
CDKN1A:FBXW5
CDKN1A
FBXW5
D
APBB1IP:FBXO21
APBB1IP
FBXO21
E
SOX12 282 TSHFEFPDYCTPEVTEMIAGDWR 304
CCND1 276 EEEEEVDLACTPTDVRDVDI--- 295
CCND2 270 KSEDELDQASTPTDVRDIDL--- 289
CCDN3 273 SSSQGPSQTSTPTDVTAIHL--- 292
. .** . :
G
APBB1IP 1 MGESSEDIDQMFSTLLGEMDLLTQSLGVDTLPPPDPN 37
EID1 160 ----------AFIEELFSLMVVN------------- 172
EID2 209 ----------TFTIALTASEVIN------------- 221
* * ::.
F
S283
I299
C291
V296
T292
P293
SOX12
H
L21
L22
L16
T23
F12

Figure S6: Predicted Aligned Error (PAE) plots for select substrate-E3 models and alignment of *AlphaFold-Multimer* annotated degron motifs to known degrons motifs, related to Figure 6.

A-D) PAE plots of E3-substrate pairs modeled using AlphaFold-Multimer. Plots are shown for each of 5 individual models generated per E3-substrate pair. Plots are provided for (**A**) PTTG1:FZR1, (**B**) GATA2:FBXL16, (**C**) CDKN1A:FBXW5, and (**D**) APBB1IP:FBXO21, which correspond to **Figure 6A-D** respectively. In each set of plots, quadrants corresponding to either the substrate or the E3 are indicated on the y-axis. Regions in the substrate that are predicted to interact with the E3 are colored blue in the lower left quadrant, and vice versa in the upper right quadrant. For all pairs, blue triangles in the Model 1 panels indicate putative degrons displayed in **Figure 6**. **E)** Alignment of the putative degron region of SOX12 and previously annotated AMBRA1-specific degron motifs in human D-type cyclins. The conserved TP motif through which AMBRA1 recognizes D-type cyclins is highlighted in red. Conserved residues (*), conservative substitutions (:), and semi-conservative substitutions (.) are indicated below the alignment. **F)** Visualization of the predicted AMBRA1 (purple) and SOX12 (tan) interaction interface with side chains of residues with any level of conservation from the alignment in panel **E** shown. **G)** Alignment of the putative degron region of APBB1IP against previously annotated FBXO21-specific degrons in EID1 and its paralog EID2. **H)** Visualization of the predicted FBXO21 (blue) and APBB1IP (red) interaction interface with side chains displayed of residues with any level of conservation from the alignment in panel **G**.

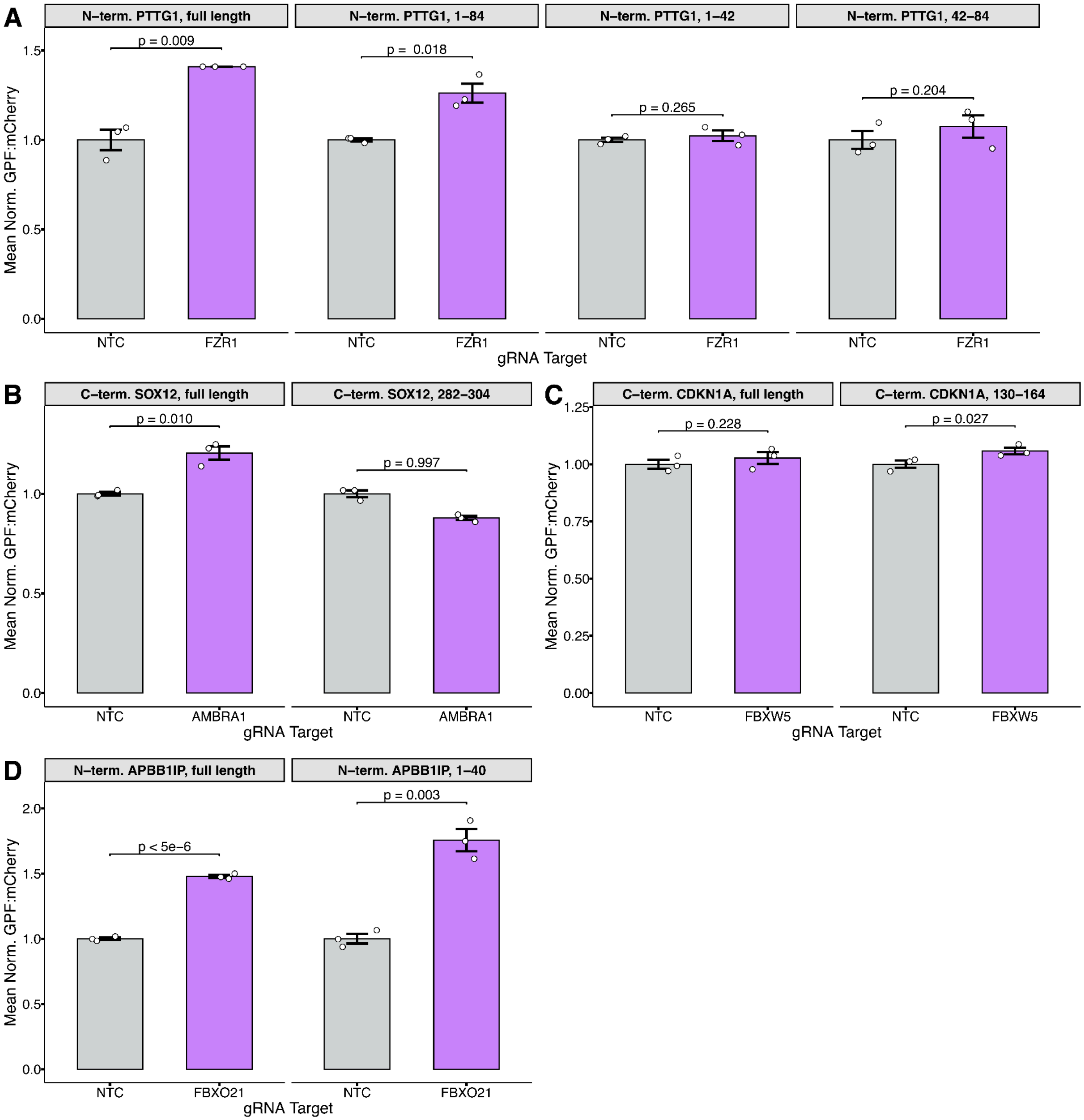

Figure S7: Assessment of E3-specific proteolysis of *AlphaFold*-predicted degrons, related to Figure 6.

NTC-normalized GFP:mCherry ratios as measured by flow cytometry are presented for *AlphaFold-Multimer* nominated degrons presented in **Figure 6**. Panels **A-D** correspond to **Figure 6A-D** respectively. Cells were transfected with the indicated combinations of GFP:mCherry reporter and gRNA expression plasmid. **A)** GFP:mCherry ratios measured in cells expressing various PTTG1 reporters and either an NTC or FZR1 gRNA plasmid. **B)** GFP:mCherry ratios measured in cells expressing various SOX12 reporters and either an NTC or AMBRA gRNA plasmid. **C)** GFP:mCherry ratios measured in cells expressing various CDKN1A reporters and either an NTC or FBXW5 gRNA plasmid. **D**) GFP:mCherry ratios measured in cells expressing various APBB1IP reporters and either an NTC or FBXO21 gRNA plasmid. Significance was calculated using a one-sided t-test.
